# An Embedded Fragment Method for Molecules in Strong Magnetic Fields

**DOI:** 10.1021/acs.jctc.2c00865

**Published:** 2022-11-22

**Authors:** Benjamin T. Speake, Tom J. P. Irons, Meilani Wibowo, Andrew G. Johnson, Grégoire David, Andrew M. Teale

**Affiliations:** † School of Chemistry, University of Nottingham, University Park, Nottingham, NG7 2RD, United KIngdom; ‡ Univ Rennes, CNRS, ISCR (Institut des Sciences Chimiques de Rennes)-UMR 6226, F-35000 Rennes, France; ¶ Hylleraas Centre for Quantum Molecular Sciences, Department of Chemistry, University of Oslo, P.O. Box 1033 Blindern, N-0315 Oslo, Norway

## Abstract

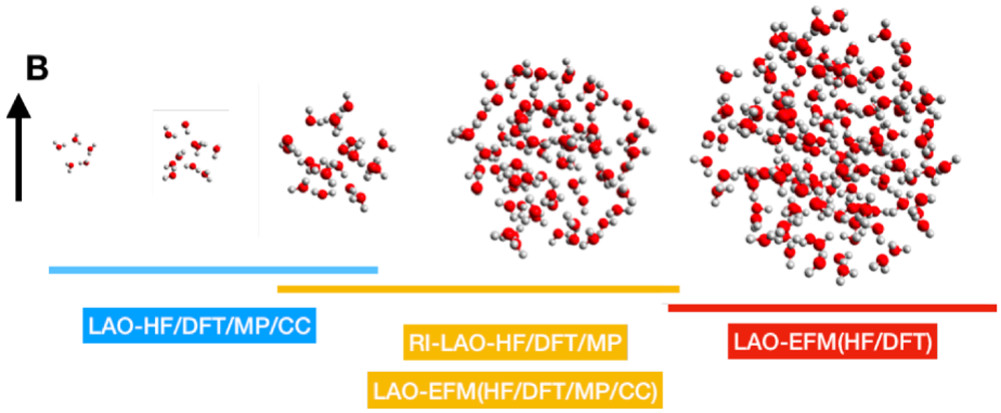

An extension of the embedded fragment method for calculations on molecular clusters is presented, which includes strong external magnetic fields. The approach is flexible, allowing for calculations at the Hartree–Fock, current-density-functional theory, Møller–Plesset perturbation theory, and coupled-cluster levels using London atomic orbitals. For systems consisting of discrete molecular subunits, calculations using London atomic orbitals can be performed in a computationally tractable manner for systems beyond the reach of conventional calculations, even those accelerated by resolution-of-the-identity or Cholesky decomposition methods. To assess the applicability of the approach, applications to water clusters are presented, showing how strong magnetic fields enhance binding within the clusters. However, our calculations suggest that, contrary to previous suggestions in the literature, this enhanced binding may not be directly attributable to strengthening of hydrogen bonding. Instead, these results suggest that this arises for larger field strengths as a response of the system to the presence of the external field, which induces a charge density build up between the monomer units. The approach is embarrassingly parallel and its computational tractability is demonstrated for clusters of up to 103 water molecules in triple-ζ basis sets, which would correspond to conventional calculations with more than 12 000 basis functions.

## 1 Introduction

Molecular clusters comprising a large number of noncovalently interacting monomers are often used to computationally model the structure of liquids and provide a means to study solvation through molecular modeling.^1−4^ However, the computational scaling of modern electronic structure methods, typically high-rank polynomial, represents a significant restriction to the size of system that may be studied and the level of methodology that may be used for such applications.^5,6^ Many approaches to overcoming the limitations on system size that can be studied have been developed; these include the introduction of approximations such as density fitting^7,8^ or the chain-of-spheres approximation,^9^ embedding methods in which certain parts of the system are treated with a higher level of theory embedded in a more approximate treatment for the rest of the system^10−12^ and fragmentation approaches in which the system is treated as a set of smaller subsystems from which the results are combined, yielding a description of the entire system.^13,14^ Such methods have become increasingly popular as the subsystem calculations are readily parallelizable while the limited size of each individual calculation allows higher level methods to be applied to the system.^15^

In recent years, there has also been a growing interest in the behavior of electronic systems in the presence of strong magnetic fields.^16−30^ Much of this has been examining the effects of magnetic fields stronger than those accessible in the laboratory but which are found to exist on the surface of white dwarf stars;^31,32^ extensive study of atoms under such conditions has been essential for identifying the spectra originating from these stellar objects.^33−35^ With the development of electronic structure packages that incorporate the nonperturbative treatment of strong magnetic fields,^36−40^ it has been possible to examine the behavior of small molecules and probe the nature of molecular bonding in these conditions using a range of electronic structure methods including Hartree–Fock theory,^16,29^ configuration interaction,^41^ coupled-cluster theory,^20^ equation of motion coupled-cluster theory,^24^ and current-density functional theory.^23,26,28,30^

While the focus of modeling electronic systems in strong magnetic fields has been on atoms and small molecules, many experimental studies have suggested that an external magnetic field can create a measurable change in the properties of bulk liquid structures.^42−51^ Furthermore, the use of a magnetic field to alter the properties of liquids within various industrial applications is well-documented.^52−54^ The rationale behind these changes, however, and in some cases the nature of the changes, is still a topic of debate; methods with which these effects can be computationally modeled in a rigorous manner have been limited. The relatively recent development of electronic structure methods that incorporate a nonperturbative treatment of magnetic field effects provides new opportunities for examining the effects of magnetic fields on the bulk properties of liquids and materials.

However, the limitations on system size that can typically be studied due to the computational cost scaling are even more restrictive when an external magnetic field is considered. This is primarily because the wave function is generally no longer real but complex with a nonzero imaginary component. Not only does this require complex arithmetic in the numerical implementation of electronic structure methods, but also there is a reduction of symmetry since complex-conjugation symmetry is lost; as a result, nonperturbative treatment of an external magnetic field raises the computational cost significantly.

In the present work, the embedded fragment-based method of Hirata et al.^55^ for modeling large clusters of noncovalently bound molecules is generalized to the case of such systems in the presence of strong magnetic fields. In this context, the computational advantages of such methods are even more significant; despite recent developments allowing a more efficient implementation of electronic structure methods in strong magnetic fields,^27,56,57^ fragment-based methods currently represent the only feasible approach for treating large systems of weakly bound molecules and examining the effects of magnetic fields on their bulk properties. Furthermore, the simple foundations of this method result in the ability to apply it to the full range of electronic structure methods generalized for systems in strong magnetic fields as described above.

In this work, the first implementation of an embedded fragment method generalized to the case of an external magnetic field is presented; this is developed within the Quest code,^39^ building on the efficient implementation of a range of methods including Hartree–Fock theory, current-density functional theory, Møller–Plesset perturbation theory and coupled cluster theory for systems in these environments. The approach is applied to the study of water clusters in the presence of strong magnetic fields, using this approach to study the effects of the field on the hydrogen bonding between water molecules in clusters of varying size.

This work is organized as follows: the theory of fragment-based approaches for weakly interacting molecular clusters and the particular method used in this work is summarized in Section 2.1. An overview of the theory underlying the development and implementation of electronic structure methods in strong magnetic fields is then given in Section 2.2, followed by a brief description of the considerations required for density functional theory under these conditions in Section 2.3. The computational methodology employed in this work is described in Section 3, with the results and discussion following in Section 4; the validity of this approach in the presence of external fields is demonstrated for small clusters in Section 4.1, with its computational scaling properties described in Section 4.2 and the effects of external magnetic fields on the strength of hydrogen bonding in large water clusters discussed in Section 4.3. Finally, some conclusions from this work and directions for future investigation are given in Section 5.

## 2 Background and Theory

### 2.1 The Embedded Fragment Method

For a large molecular system that comprises many discrete fragments, each internally bound covalently but interacting with each other in a noncovalent manner, the energy *E* may be represented as the sum of fragment energies according to the many body expansion (MBE),^58−62^
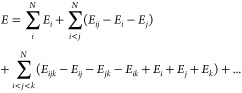
1where *E*_*i*_ is the energy of the fragment (hereafter referred to as monomer) *i*, *E*_*ij*_ is the energy of the dimer comprising monomers *i* and *j*, with the trimer energy *E*_*ijk*_ and higher-order terms similarly defined. The MBE in eq 1 becomes exact for a system of *N* monomers when extended to include *N*-body terms; however, evaluating the energy in this way is not computationally advantageous unless the series can be truncated at some lower order with minimal loss of accuracy.

The simplest approximation to the energy is obtained by truncating eq 1 to second-order as
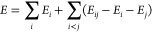
2including the monomer energies and pairwise-additive corrections for the interaction between monomers. While this approach typically recovers most of the interaction energy, higher-order terms often still contribute significantly to the interaction energy with their omission resulting in large errors in the second-order MBE energy.^14,63,64^ For molecular clusters comprising monomers with nonzero electronic dipole moments, such as the water clusters considered in this work, third- and fourth-order terms in the MBE contribute significantly to the energy,^63,65,66^ with even fifth-order terms making a non-negligible contribution for some larger clusters.^67^

The number of terms in the *k*th-order expansion of the MBE for a cluster comprising *N* monomers scales as ; the cost of evaluating the energy with eq 1 quickly becomes unfeasible as higher-order terms are considered. Furthermore, with such a large number of individual calculations contributing to the total energy when higher-order terms are considered, each must be converged to within a tight threshold to minimize the accumulated loss of precision in the cluster energy.^15,68,69^

The origin of these nonpairwise additive contributions to the energy of dispersion-bound clusters, requiring the extension of the MBE beyond two-body terms, has been the subject of investigation for many decades; three-body contributions to the interaction energy of atomic clusters were first characterized by Axilrod-Teller^70^ and Muto^71^ in 1943. Much of the subsequent work has considered the many-body contributions in the interaction between molecules in water clusters,^1,2,14,55,58,72,73^ because of the obvious biochemical significance of these systems.

The most significant nonpairwise additive contributions to the energy of weakly interacting water clusters can be identified by considering the different many-body components of the energy.^59^ Since the electron density of neutral species decays exponentially with distance *r*, the same is true for the exchange interaction^74−76^ and therefore its contribution to the nonadditive interaction will be small and only arise at small separation between monomers. Similarly, correlation interactions decay rapidly as *r*^–6^ and are thought to be near-pairwise additive for water clusters.^67,77,78^ By contrast the Coulomb interaction exhibits a much slower asymptotic decay, to first order decaying as *r*^–3^ for neutral systems.^79,80^ As a result, each monomer has a non-negligible interaction with potentially a large number of other monomers in the cluster; the resulting polarization effects^81−83^ dominate the nonpairwise additive contribution to the energy and become more significant with increasing cluster size.^68,84^

There are many different approaches that have been developed to make the MBE more computationally tractable by accelerating its convergence, such that sufficient accuracy can be achieved even when the series is truncated to a lower order; recent reviews can be found in refs (13), (62), and (85). In many such methods, an external potential is introduced to the Hamiltonian for each subsystem representing the electrostatic potential of the rest of the system, thus introducing many-body polarization effects to the calculation of even the monomer and dimer energies.

Many different methods exist for representing the electrostatic potential of the other monomers in the Hamiltonian of each subsystem. In the electrostatically embedded MBE approach of Dahlke and Truhlar, each monomer is represented by point charges at the nuclei but with the partial atomic charges for the molecule, either calculated in isolation for each monomer or self-consistently over the entire system.^73,86−88^ Perhaps the most well-known method, however, is the fragment molecular orbital (FMO) method of Kitaura and co-workers.^89−91^ For this, each subsystem is embedded in the Coulomb potential of the other monomers in the system, with the effective Hamiltonian for a general subsystem η comprising monomers {*i*_1_, ..., *i*_*m*_} taking the form
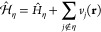
3where  is the Hamiltonian of η in isolation and *v*_*j*_(**r**) the Coulomb potential of the *j*th monomer
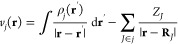
4with electron density ρ_*j*_ and nuclei with charges *Z*_*J*_ located at **R**_*J*_. This Coulomb potential is determined self-consistently by iterating the density of each monomer in the presence of the embedding potential until the densities of all the monomers converge; this is often referred to as the self-consistent charge procedure. The energy of each term in the MBE is then evaluated in the presence of the self-consistent Coulomb potential of the rest of the system by obtaining solutions to the Schrödinger equation with the Hamiltonian of eq 3,
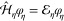
5and the MBE energy computed from the  with eq 1. This approach constitutes a full many-body treatment of the Coulomb interaction; other interactions are considered to the level at which the MBE is truncated, where second-order,^89^ third-order,^92^ and fourth-order^93^ truncation of the MBE are often employed. The FMO method has been applied to advanced *ab initio* methods such as coupled-cluster theory^94^ and extended to the calculation of excitation energies with time-dependent density functional theory.^95^

In this work, a simplification to the FMO method first proposed by Hirata et al.^55^ is employed. In this approach, the multipole expansion for the Coulomb potential of eq 4 is considered due to the localized nature of the charge distributions in each monomer
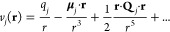
6where *q*_*j*_, **μ**_*j*_, and **Q**_*j*_ are the electrostatic monopole, dipole, and quadrupole moments of the *j*th monomer, respectively. For neutral monomers the first term vanishes and the leading order term is simply the dipole potential,
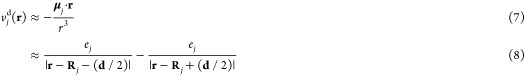
in which the dipole potential is modeled by a pair of point charges (*e*_*j*_, −*e*_*j*_) separated by a distance *d* and centered at **R**_*j*_, the position at which the nuclear dipole moment of the *j*th monomer vanishes. In practice, the dipole length is set to a value of 0.01 bohr such that |**r**| ≫ |**d**|, in the limit of which the representation of the potential in eq 8 equals that in eq 7. Following the FMO method, the potential is optimized self-consistently at the monomer level by updating *e*_*j*_ and **d** such that *e*_*j*_**d** = **μ**_*j*_ at each iteration, until the values of *e*_*j*_ are converged^55^ to within a given threshold (in this work, a value of 10^–4^ a.u.). The converged potential is then included in the Hamiltonian for evaluation of the terms in the MBE in an analogous manner to that for FMO in eq 4. However, this form of potential does not directly include the effect of magnetic induction; this is expected to be negligible except at very high fields and in this work the efficacy of the embedding potential given in eq 8 at high fields will be tested.

There are clear advantages to this approach: it is significantly less computationally intensive than the original FMO method, in which the full Coulomb potential is required at each iteration. However, the leading order term of the embedding potential remains properly described; this approach has been shown to yield a highly accurate treatment for weakly bound molecular clusters.^55^

One further consideration that must be made in the evaluation of cluster energies by fragmentation methods is the effect of basis-set superposition. This arises due to the overlap of basis functions on nearby monomers stabilizing the energy of the corresponding dimer in a way that does not occur if the monomers are well-separated and their basis functions do not overlap.^96−99^ This so-called basis-set superposition error (BSSE) is not an error in the calculation of dimer energies themselves, but instead an inconsistency in the treatment of dimers, based on how well-separated their constituent monomers are, that can introduce errors into the MBE.^100^

There exist various treatments for removing the BSSE from the interaction energies of noncovalently bound molecular clusters^101−105^ based on the counterpoise correction of Boys and Bernardi, which estimates the BSSE contribution to the energy associated with each monomer in a dimer to be the difference between the energy of the monomer evaluated in its own basis and its energy evaluated in the dimer basis.^101^ In the present work, the generalization of the Boys–Bernardi counterpoise correction to fragments with an embedding potential of the form in eq 8 is used to correct for the BSSE;^106^ for the second-order MBE of eq 2, the BSSE-corrected energy of the cluster  is evaluated as
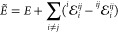
9where the notation  denotes the energy of monomer *i* evaluated in the basis of subsystem *a* and in the embedding potential of all monomers excluding those in subsystem *b*, where *i* ∈ *a*,*b*.

### 2.2 Systems in Strong Magnetic Fields

In the present work, the many-body expansion outlined in the previous subsection is applied to study the effects of strong magnetic fields, up to a strength of 1*B*_0_ = ℏe^–1^*a*_0_^–2^ = 2.3505 × 10^5^ T, on intermolecular interactions in noncovalently bound molecular clusters. Under these conditions, magnetic interactions can become comparable to Coulomb interactions in strength, resulting in an exotic chemistry while requiring a fully nonperturbative treatment of the field in the calculation of the electronic structure.

The nonrelativistic electronic Hamiltonian in the presence of a uniform magnetic field **B** can be expressed in atomic units as
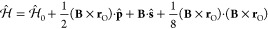
10in which the zero-field Hamiltonian is denoted , the canonical momentum operator , the spin operator , and the position relative to an arbitrary gauge origin **O** written as **r**_O_ = **r** – **O**. Since ∇·**B** = 0 according to Gauss’ law for magnetism, there exists a vector field known as the magnetic vector potential **A** for which **B** = ∇ × **A**; however, this relation is not uniquely satisfied and can only be defined to within a particular gauge; in the present work, the Coulomb gauge for which ∇·**A** = 0 is employed. For a uniform magnetic field **B**, **A** may be expressed in terms of the gauge origin **O** as
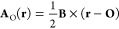
11for which a translation of **O** to some new position **O**′ transforms the vector potential as
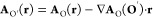
12This transformation of the gauge is associated with a unitary transformation of the Hamiltonian and compensating unitary transformation of its eigenfunctions, given respectively by
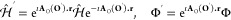
13while the observables of the system such as the energy and the charge density remain invariant to the gauge transformation. The dependence on the gauge origin that is introduced to the wave function in eq 13 cannot be reproduced by a finite basis of Gaussian or Slater functions. However, explicit inclusion of the gauge origin in the basis functions themselves permits the gauge-dependent wave functions to be represented in a finite basis. This approach is taken in the construction of London atomic orbitals (LAOs),^80,107^ which comprise a standard Gaussian-type basis function φ multiplied by a complex phase factor containing the gauge origin,
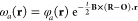
14Using a basis of LAOs yields wave functions that exhibit the correct behavior to first order, with respect to the magnetic field and rigorously gauge-origin invariant observables, allowing the behavior of electronic systems in arbitrary field strengths to be examined nonperturbatively.^16^

In this work, the energies of molecular clusters in the presence of magnetic fields are evaluated using several methods: Hartree–Fock (HF), second-/third-order Møller–Plesset perturbation theory (MP2/3), coupled-cluster singles and doubles (CCSD) and Kohn–Sham density functional theory (KS DFT) with several exchange–correlation functionals.

The HF, MP2/3, and CCSD methods are relatively unchanged when applied to systems in strong magnetic fields, with the main differences arising in the evaluation of molecular integrals due to the use of LAOs^16,27^ and ensuring complex-conjugate symmetry is properly respected in the implementation of post-HF methods to treat correlation.^20,24^ However, the adaptation of KS DFT to these conditions requires additional considerations; these are briefly outlined in the following subsection.

### 2.3 Current Density Functional Theory

In the presence of an external magnetic field, the additional field-dependent terms present in the Hamiltonian of eq 10 has the result that an electronic system under these conditions cannot be described by just the charge density alone as is the basis for zero-field DFT.^108,109^ The universal density functional must include either a direct dependence on the magnetic field (a formulation called magnetic field DFT (BDFT))^110,111^ or dependence on the magnetically induced current density (the current DFT (CDFT) formalism).^18,112,113^

The present work employs the Vignale–Rasolt form of the latter, in which the universal density functional  is dependent on the charge density ρ and the paramagnetic current density **j**_p_ and the energy *E* is dependent on the scalar potential *v* and vector potential **A**. This may be cast in the convex-conjugate formalism of Lieb^114^ by defining a scalar potential *u* = *v* +  and associated energy functional , which is, by construction, concave in the potential and related to  by the convex conjugate relationships:^18,115^
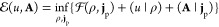
15
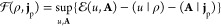
16in which (*u*|ρ) = ∫*u*(**r**)ρ(**r**) d**r** and (**A**|**j**_p_) = ∫**A**(**r**) ·**j**_p_(**r**) d**r**. The Kohn–Sham decomposition of  yields^25,109^
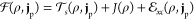
17with noninteracting kinetic energy , Coulomb repulsion energy *J*(ρ) and the exchange–correlation energy . The KS CDFT equations are given by
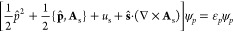
18which may be solved to yield one-particle KS orbitals ψ_*p*_ and energies ε_*p*_. The noninteracting auxiliary system in KS CDFT has a charge density and paramagnetic current density defined in terms of ψ_*p*_ with spin σ as
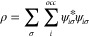
19
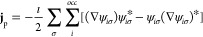
20to reproduce the charge and paramagnetic current densities of the physically interacting system, respectively. Therefore, the KS potentials (*u*_*s*_, **A**_*s*_) are defined as
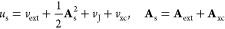
21with *v*_ext_ and **A**_ext_ being the physical external potentials arising due to the nuclei and the applied field, respectively, *v*_J_ the Coulomb potential and the remaining terms the scalar and vector exchange–correlation potentials, defined as
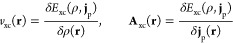
22The functional form that the **j**_p_ dependence should take is not known; however, it has been found that meta-generalized gradient approximations (mGGAs) that are dependent on the noninteracting kinetic energy density are reliable and accurate for systems in strong magnetic fields.^23^ In CDFT, the noninteracting kinetic energy density is modified to ensure such exchange–correlation functionals remain gauge-origin invariant; in this work, the modification of Dobson^116,117^ and Becke,^118^
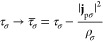
23is substituted into the Tao–Perdew–Staroverov–Scuseria functional, denoted cTPSS,^119,120^ thus permitting its uniform application to systems in the presence of increasing field strengths.

Both for HF/MP2/CCSD and CDFT, the computational cost when using LAOs is significantly greater than that of using GAOs with no magnetic field applied; every floating point operation involving the basis functions must be evaluated using complex arithmetic, while the permutational symmetry of the two electron integrals is reduced from 8-fold to 4-fold.^16,27^ There have been many recent developments in computational approaches for using LAOs; these include the development of efficient algorithms for the evaluation of the integrals^27^ and their derivatives,^28^ the application of the resolution of the identity (RI) approximation^56^ and the Cholesky decomposition of the two-electron integrals.^57^ Despite these developments, the size of the system that can be considered in strong magnetic fields using LAOs remains much smaller than those that can be studied using standard Gaussian basis sets with highly optimized codes.

It is in this context that the MBE presents a highly interesting opportunity to study larger systems in strong magnetic fields; the generalization of the second-order MBE using the dipole embedding potential of Hirata et al.^55^ to systems in strong magnetic fields is developed and implemented in the present work, to enable, for the first time, the study of intermolecular interactions in weakly bound molecular clusters under extreme conditions.

## 3 Computational Methodology

In the present work, the second-order MBE of eq 2 with the dipole embedding potential of eq 8, hereafter referred to as the embedded fragment method (EFM), has been implemented into the Quest quantum chemistry code,^39^ building on the extensive infrastructure for electronic structure calculations in strong magnetic fields developed in the program. The message passing interface (MPI) was utilized to parallelize the EFM calculations.^121^ Once the embedding field has been determined self-consistently all monomer and dimer calculations can be performed independently in the presence of this field, with minimal communication. This results in an approach that is embarrasingly parallel, with a consistent reduction in the time taken for the calculation with increasing computational resource; this is discussed in Section 4.2.

In this work, a range of electronic structure methods and basis sets were employed for the study of different cluster sizes; the water trimer was studied at the HF, MP2, MP3, CCSD, and CDFT levels with the BLYP and cTPSS functionals in the aug-cc-pVDZ basis set of Dunning,^122^ while water clusters with between 3 and 103 monomers have been considered at the HF, MP2 and CDFT/cTPSS levels in both the aug-cc-pVDZ and aug-cc-pVTZ basis sets.^123,124^ In the case of the largest water clusters, the RI approximation has been employed with auxiliary basis sets automatically generated using the AutoAux method of Stoychev et al.,^125^ which constructs a set of fitting functions to span the product space of the orbital basis.

A particular adaptation of the EFM employed in the present calculations is the construction of initial guess densities for the dimers from a superposition of monomer density matrices, generated during the determination of the monomer energies step. This improves the efficiency of the dimer calculations, consistently providing a more accurate initial guess, including the embedding field contributions, compared to standard methods for the generation of initial guess densities such as from the core Hamiltonian or from a superposition of atomic densities.

### 3.1 Determination of Water Cluster Geometries

The geometries of the smaller water clusters considered here, with between 3 and 10 monomers, were taken from a set of benchmark data for more than 70 low-lying water clusters,^126^ where the geometries were determined at the MP2 level with a combination of the aug-cc-pVTZ and cc-pVTZ basis sets for the oxygen and hydrogen atoms, respectively. For purposes of comparison, however, the water trimer used for the analysis presented in Tables 1 and 2, was optimized at the HF/aug-cc-pVDZ level to reproduce the geometry used in ref (55).

**Table 1:** Error in the Total Water Trimer Energy Evaluated Using EFM, Relative to Conventional Evaluation at Increasing Magnetic Field Strengths (Oriented as Shown in Figure 5) in the aug-cc-pVDZ Basis for a Range of Electronic Structure Methods

	Error in Total Water Trimer Energy (kcal mol^–1^)				
|**B**|/*B*_0_	0.00	0.10	0.20	0.30	0.40
HF	–0.14	–0.12	–0.19	–0.24	–0.52
BLYP	–0.56	–0.49	–0.57	–0.61	–1.41
cTPSS	–0.58	–0.54	–0.61	–0.70	–1.37
MP2	–0.37	–0.28	–0.30	–0.32	–0.68
MP3	–0.36	–0.27	–0.29	–0.31	–0.63
CCSD	–0.37	–0.28	–0.29	–0.31	–0.64

**Table 2:** Trimer Interaction Energy at Increasing Magnetic Field Strengths (Oriented as Shown in Figure 5) in the aug-cc-pVDZ Basis for a Range of Electronic Structure Methods

	Trimer Interaction Energy (kcal mol^–1^)				
|**B**|/*B*_0_	0.00	0.10	0.20	0.30	0.40
HF	–11.9	–12.8	–14.8	–16.4	–15.6
BLYP	–12.8	–14.0	–16.7	–18.7	–18.3
cTPSS	–13.7	–14.7	–17.2	–18.9	–18.4
MP2	–15.9	–16.8	–19.1	–20.9	–20.4
MP3	–15.1	–16.0	–18.2	–19.8	–19.1
CCSD	–15.3	–16.2	–18.4	–20.0	–19.2

The geometry of the largest water cluster, which consists of 103 monomers, was taken from a snapshot of a molecular dynamics (MD) simulation. The water cluster was simulated in a cubic simulation box of dimension 30 Å and described with the TIP3P water model.^127,128^ The MD simulation was performed using the NVT ensemble with a constant temperature of 300 K for a duration of 150 ps. The MD simulation was performed in the absence of an external magnetic field–yielding zero-field geometries analogous to those obtained for the smaller water clusters. This simulation was performed using the DL_POLY software package.^129^

### 3.2 Short Range Potential Attenuation

As described in Section 2.1, the embedding potential used in this work is a first-order truncation of the multipole expansion for the Coulomb potential of each monomer, which is represented by only the dipole potential term. This generally results in very little loss of accuracy since the monomers are relatively well-separated when noncovalently bound and therefore the Coulomb potential of other monomers in regions of nonzero electron density for a given subsystem will be dominated by the dipole potential term.^55^

However, for some geometries of water clusters considered in this work, there were several monomers in sufficiently close proximity that the dipole potential did not accurately represent the Coulomb interaction between them. The absence of the significant contributions from higher-order terms at short-range results in an unphysical potential in this region, the interaction of which with the charge density of other subsystems can make their densities more difficult to converge and the resulting errors propagate through the MBE. This effect is particularly apparent when CDFT is employed due to the more diffuse charge density—an artifact of the delocalization error arising from using approximate exchange–correlation functionals.^130^

**Figure 1:**
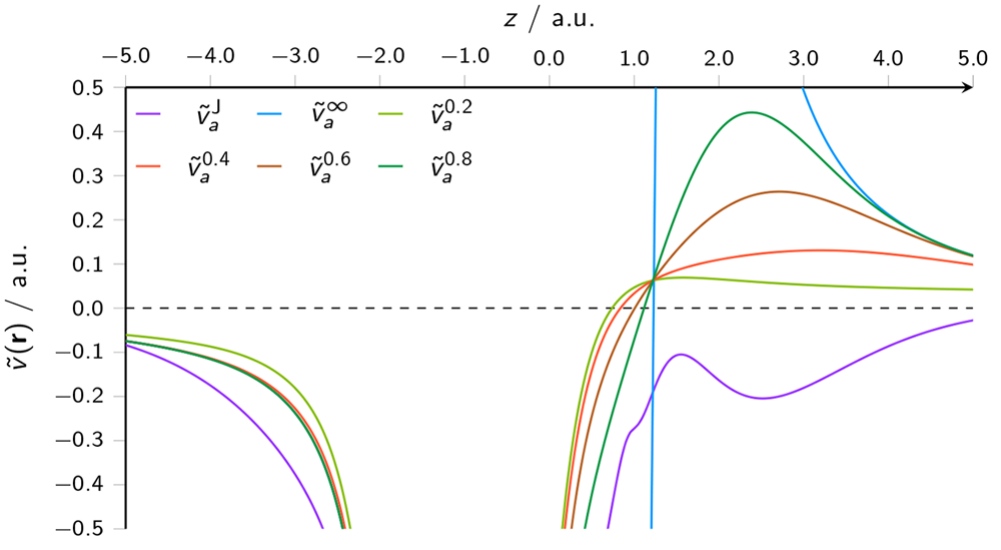
Effective potential for a water molecule, in the presence of another water molecule at a distance of 2.0 bohr, constructed from the full Coulomb potential  and the attenuated dipole potential , according to eqs 25 and 26, respectively.

**Figure 2:**
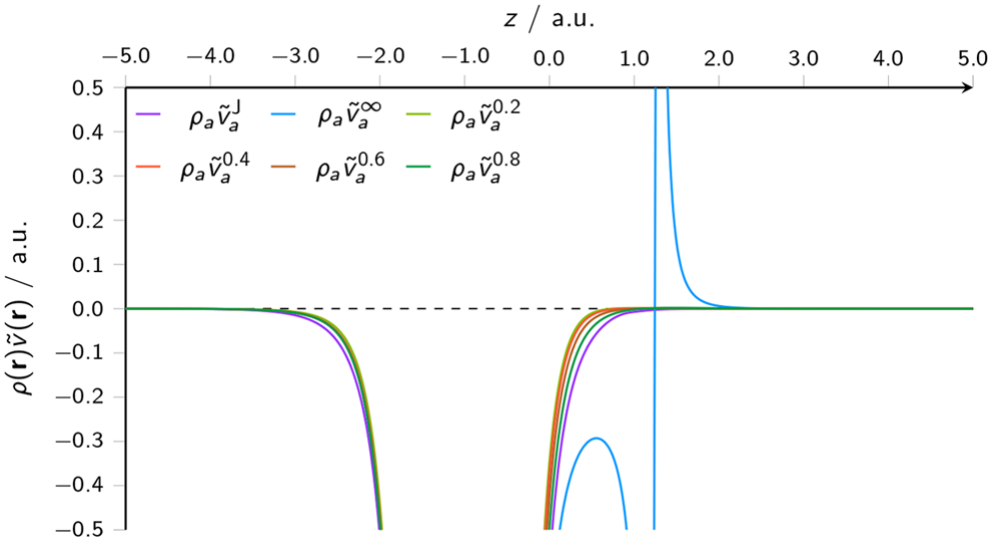
Effective potential for a water molecule multiplied by its density ρ_*a*_, in the presence of another water molecule at a distance of 2.0 bohr, constructed from the full Coulomb potential  and the attenuated dipole potential , according to eqs 25 and 26, respectively.

The errors at short-range in the multipole expansion are well-known and strategies for addressing these problems using damping functions have been discussed for example in ref (131). Furthermore, the limitations of the dipole potential at short-range have been discussed in ref (106), in which it is augmented by an electrostatic potential represented by several point charges for each monomer, the position and partial charges of which are optimized such that the resulting potential best matches the Coulomb potential of the monomer. In the present work, an alternative approach is proposed; the dipole potential of each monomer is attenuated at short-range with the error function, as
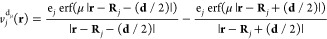
24in which μ is the attenuation parameter: as μ → 0, erf(μ) → 0 while as μ → *∞*, erf(μ) → 1. The effect of attenuation on the potential following eq 24 can be seen by considering the effective Coulomb potential of water molecule *a* in the presence of another water molecule *b* at a distance of 2.0 bohr. Therefore, the effective potential in  may be represented by the difference between the Coulomb potential of the two molecules combined *v*_*ab*_ and that of the second water molecule *v*_*b*_,
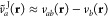
25in which the Coulomb potentials are as defined in eq 4. This may be compared with the effective potential  constructed from the Coulomb potential of *a* and the dipole potential of *b*, as defined in eq 24, to give
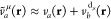
26The potentials of eqs 25 and 26 are plotted in Figure 1, for a range of attenuation parameters. It can be seen clearly how the dipole potential departs significantly from the Coulomb potential in the vicinity of water molecule *b* at this separation, while the attenuation at short range significantly reduces the potential in this region. In Figure 2, the product of these potentials with the charge density of the water molecule ρ_*a*_ is plotted; this shows how attenuation of the potential in the regions that contribute meaningfully to the energy of the molecule has a significant effect, bringing the effective potential closer to the Coulomb potential that it is intended to model.

**Figure 3:**
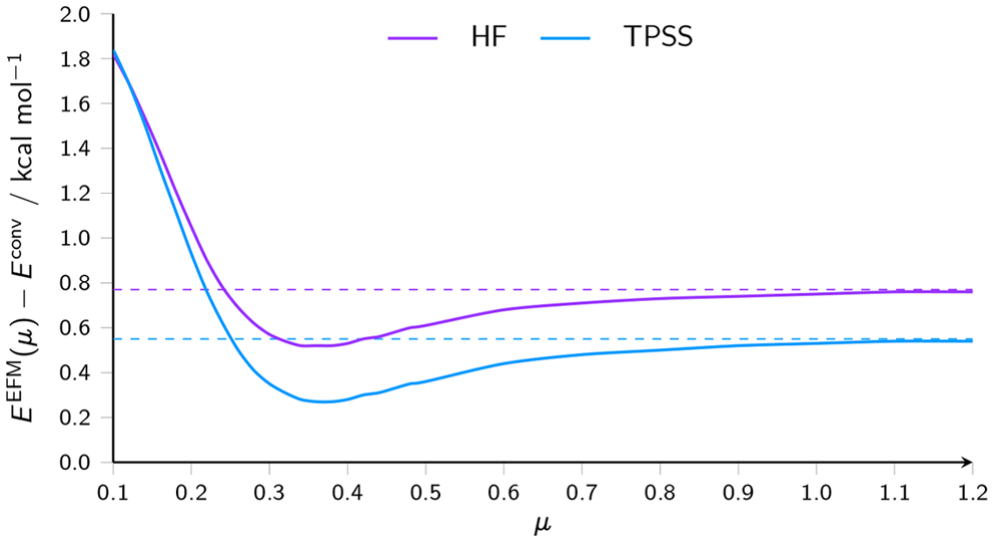
Error in the energy of a water trimer, evaluated with the EFM method relative to a conventional calculation, as a function of embedding potential attenuation parameter μ (given in units of kcal mol^–1^), calculated using HF and TPSS.

In both figures, the dipole potential is plotted for several attenuation parameters; even though these values of μ result in significant short-range attenuation, it can be seen particularly in Figure 1 that values of this order are required to remove the unphysical oscillatory feature around the origin of the dipole. Even with a significant attenuation of the potential, however, the error in the energy, with respect to a conventional calculation, rapidly approaches that with the unattenuated potential. This can be seen in Figure 3, which shows the change in the error in the energy from the EFM method relative to a conventional calculation, with respect to the attenuation parameter μ. With both HF and TPSS, the error begins to approach that of the unattenuated EFM calculation already at μ ≈ 1, whereas, for 0.25 ≤ μ ≤ 1.00, the error obtained by using the attenuated potential is lower than that of the unattenuated calculation. We have confirmed that a similar trend occurs for water clusters of up to 10 monomers in a variety of conformations.

## 4 Results and Discussion

### 4.1 The Embedded Fragment Method in Strong Magnetic Fields

**Figure 4:**
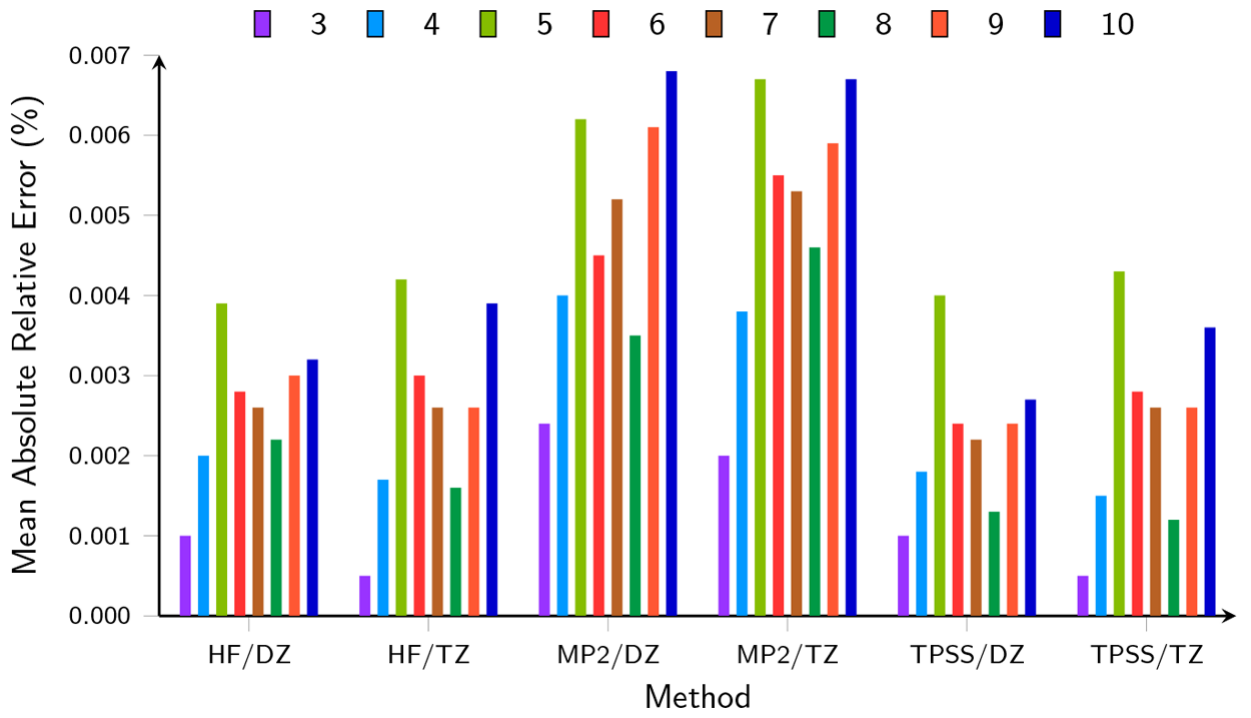
Mean absolute relative error (%) in the total energy calculated by EFM, compared to the corresponding conventional approaches, for water clusters with between 3 and 10 monomers at different levels of theory with both the aug-cc-pVDZ (DZ) and aug-cc-pVTZ (TZ) basis sets.

We commence by assessing the accuracy of the present implementation by comparing the energy of small water clusters evaluated using the EFM with that given by conventional single-point calculations on the entire clusters at zero field. In this analysis, water clusters with between 3 and 10 monomers are considered; the mean absolute relative error is calculated across a range of conformers, with geometries taken from ref (126), for each cluster size using several different methods and basis sets. These are presented in Figure 4, while the underlying energies from which these errors are calculated may be found in the Supporting Information.

Several observations can be made from Figure 4. First, in all cases, the mean error is below 0.01%, which is consistent with those of ref (55), confirming the accuracy of the present implementation at zero field. Second, with the exception of the trimer, which has a much smaller error than other cluster sizes, the size of the error is relatively consistent with increasing cluster size. This is reassuring, given the propensity for precision errors to accumulate in MBE methods.^69^ This relative insensitivity of the error to cluster size is observed when the BSSE is taken into account as described in Section 2.1. Third, there are no significant differences in relative error with the larger aug-cc-pVTZ basis set, compared to the aug-cc-pVDZ basis, indicating the overall efficacy of the BSSE correction described in Section 2.1. The average errors are very similar between HF and TPSS, while being slightly larger with MP2.

**Figure 5:**
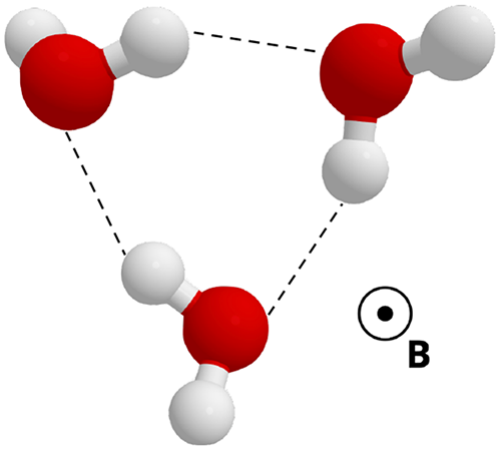
Geometry of the water trimer used in the present work, showing the relative orientation of the applied magnetic field.

With the accuracy of the present implementation established at zero field, its performance in the presence of strong magnetic fields can now be considered. Table 1 shows the error in the total energy of the water trimer evaluated using EFM relative to its energy evaluated with a conventional calculation of the entire system for several electronic structure methods with increasing magnetic field strength. For the purposes of comparison with ref (55), the BSSE correction is not included in the EFM energy when calculating the errors in Table 1 and interaction energies in Table 2. The magnetic field is applied perpendicular to the plane of the three oxygen atoms in the trimer, shown in Figure 5. The underlying data from which the errors are calculated may be found in the Supporting Information.

It can be seen in Table 1 that the errors in the energies with EFM do not change significantly from zero field up to |**B**| = 0.3*B*_0_, for each of the methods considered here; however, the errors roughly double as the field strength increases from |**B**| = 0.3*B*_0_ to 0.4*B*_0_. The sharp increase in the error as the field strength increases beyond |**B**| ≈ 0.3*B*_0_ does not have a single obvious cause; there may be several contributory factors for this observation.

One reason this method may not remain as accurate at arbitrary field strengths, at least without further modification, is due to the change that occurs in the relative significance of Coulomb interaction at very high field strengths; in the region of |**B**| ≈ 1.0*B*_0_, magnetically induced interactions become as significant as the Coulomb interaction. The embedding potential in the present work comprises the dominant term in the multipole expansion of the Coulomb potential, determined self-consistently at the monomer level. However, this may be an increasingly incomplete representation of the interaction between spatially separated charge distributions on different molecules. In addition, since this interaction is with an external field, it may be intrinsically more difficult to describe by low-order terms in the MBE. Nevertheless, Table 1 does suggest that, up to a limit, the EFM approach remains reliable for systems in relatively strong magnetic fields.

Given this, it is therefore possible to investigate the effects of increasing magnetic field strength on the clusters. In the simplest case, the changes in interaction energy, as defined by the difference between the EFM energy (*E*^EFM^) and the sum of the energies of isolated monomers (*E*^iso^), with magnetic field strength can be examined for the water cluster. Table 2 presents the interaction energy for the trimer at increasing field strengths, with a range of electronic structure methods.

It can be seen in Table 2 that, for all electronic structure methods, the interaction energy becomes more negative from zero field up to |**B**| = 0.3*B*_0_, beyond which there is an apparent decrease in the magnitude of the interaction energy. However, there is a limit to how much can be interpreted from this, since the geometry of the trimer remains fixed at the zero field geometry for all field strengths; it has been seen^28,30^ that, at the field strengths considered in Table 2, the equilibrium geometry can be significantly different to that at zero field. A more meaningful picture of the trend in interaction energy with field strength would require relaxation of the geometry to be considered, which is beyond the scope of the present study. For lower field strengths, however, the effects of geometry relaxation are expected to be much smaller and therefore examination of the change in interaction energy with field strength in the range |**B**| ≲ 0.1*B*_0_ for much larger clusters even at fixed geometries can be used to investigate changes in properties under such conditions; this is discussed in Section 4.3.

In addition to interaction energies, EFM can further be used to examine the changes in electron density within the system with field strength. In the MBE paradigm, the total electron density for a given cluster can be evaluated as^132^
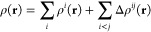
27where
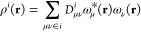
28
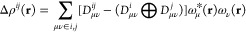
29Here, *D*^*i*^ is the density matrix of monomer *i* and *D*^*ij*^ is the density matrix of the dimer comprising monomer *i* and monomer *j*, while μ and ν are the indices of the basis functions.

**Figure 6:**
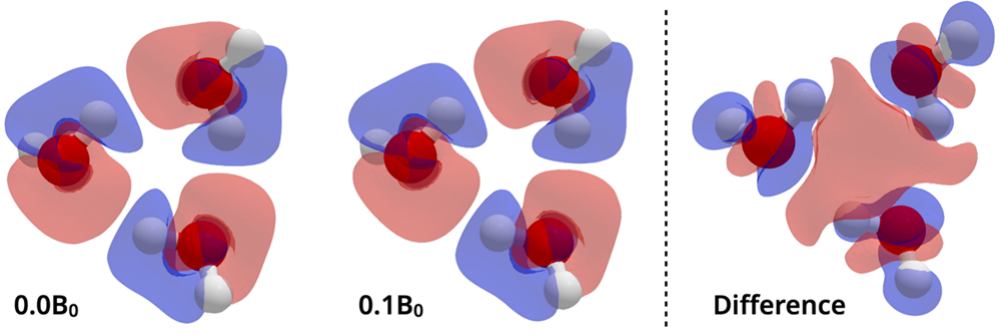
Density difference plots for a water trimer determined at two different field strengths (|**B**| = 0.0*B*_0_ and 0.1*B*_0_, oriented as shown in Figure 5) and the difference between them (right). All calculations performed using DFT with the cTPSS functional and the aug-cc-pVDZ basis set. Red indicates a buildup of electron density and blue represents a depletion.

Figure 6 shows, for the same fixed geometry water trimer, how the electron density relative to the isolated water molecules changes as the magnetic field strength is increased from zero to |**B**| = 0.1*B*_0_. It can be seen that there is a general increase in electron density in the region between the three monomers at |**B**| = 0.1*B*_0_, relative to zero field, which would indicate a strengthening of the interactions between the three molecules. However, this is not directly consistent with an increased charge density accumulation on the oxygen atom and depletion on the hydrogen atom that would be expected from an increased hydrogen bonding strength.

### 4.2 Computational Efficiency and Accuracy Considerations

Having established the validity of the EFM approach to treat small clusters in the presence of strong magnetic fields we now consider the computational scaling of the present implementation. As described in Section 2, the use of LAOs inherently raises the computational cost of all electronic structure methods, although many approaches for reducing the scaling of conventional calculations have been developed such as RI^28,56,133^ and the Cholesky decomposition.^57^

It has been shown that using the binary approximation effectively reduces the computational scaling, with relation to the system size, to , regardless of the underlying electronic structure method that is chosen. It can be further reduced to effectively  for very large clusters if a radial cutoff is introduced when calculating the dimer energies, assuming that the two-body electron correlation contributions decay much faster than the Coulomb contributions accounted for by the embedding potential.

**Figure 7:**
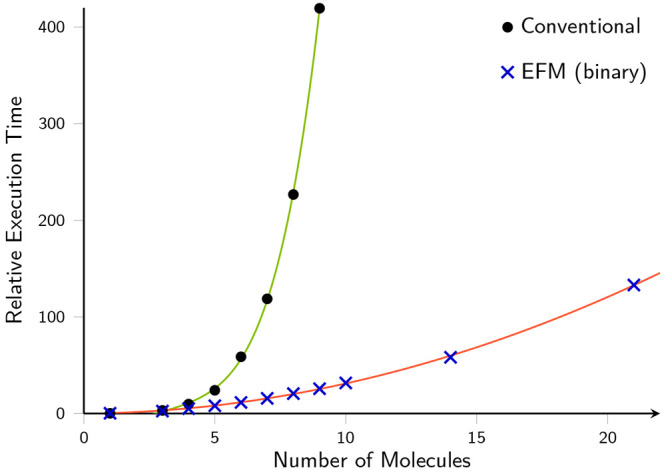
Relative CPU time required to perform LAO based RI-MP2 single-point energy calculations on a series of water clusters, with and without EFM.

This remains the case when using LAO-based electronic structure methods, since inherently the computational cost is still only dependent on a series of dimer calculations, which are individually very inexpensive, relative to a calculation on the entire system. Figure 7 demonstrates the reduction in scaling from  to the expected  for MP2 calculations, with the aug-cc-pVDZ basis set and RI approximation (the implementation of RI-MP2 used in this work is described in Appendix A), on a series of water clusters with increasing size in a magnetic field of |**B**| = 0.1*B*_0_. All execution times are measured relative to the time required for the calculation on a single water molecule.

**Figure 8:**
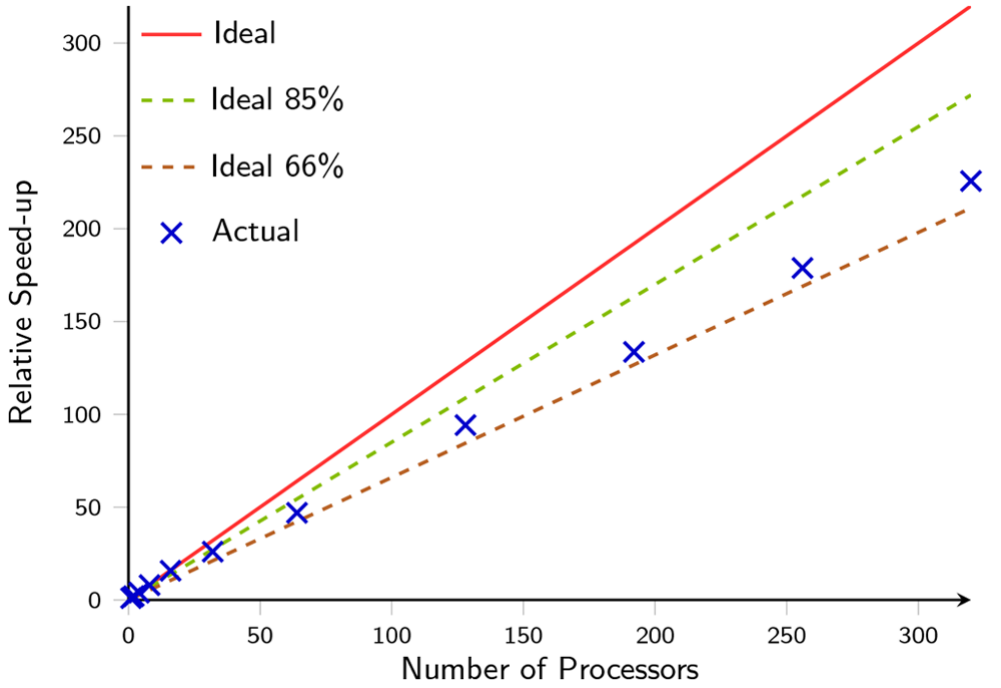
Measured acceleration when increasing the number of processors for a HF/aug-cc-pVDZ EFM calculation on a cluster consisting of 103 molecules, compared to the ideal acceleration at three different clock speeds, which account for variable frequency scaling.

This method significantly benefits from the efficient use of high-performance computer facilities, since each stage within a given calculation is inherently embarrassingly parallel. Individual monomer and dimer energy calculations can be distributed across multiple processors, decreasing the overall time required to perform the total calculation. This can be demonstrated by plotting the acceleration, , where *t*_0_ is the time taken on a single processor, achieved when the number of processors used for a single calculation is increased. This is demonstrated in Figure 8 and compared against *ideal* acceleration, in which the computation time is inversely proportional to the number of processors. To account for variable frequency scaling, two other lines have been included which show the *ideal* acceleration scaled down by 15% and 33%, in accordance with the reported clock speeds for AMD EPYC 7551 processors used in this work. This reduction accounts for the dynamic lowering of the clock speed as the number of active cores is increased.

### 4.3 Water in Strong Magnetic Fields

Following from the previous discussion, it is interesting to now consider the application of EFM to much larger water clusters, the behavior of which should approach that of water on the macroscopic scale as the size of the cluster increases. The effect of an applied magnetic field on bulk properties of systems can be relatively small, depending on the system and the strength of the magnetic field. However, many studies have shown that even weak fields can cause a measurable change in various properties of liquid water.^42−51^ The underlying mechanism by which these changes occur has not been unambiguously determined, with some studies suggesting that the external field influences the hydrogen bonding within water.^42−44,47,48,50^ However, many of these observations have proven difficult to reproduce and their physical origins remain a matter of debate.

To examine this problem computationally, either the use of very large system sizes, solvation models or periodic boundary conditions must be employed; all of these would be highly computationally expensive and, in some cases, can be difficult to generalize to the presence of an external magnetic field. EFM enables the computational study of increasingly large water clusters, in a basis of LAOs for the nonperturbative treatment of an external magnetic field, providing the possibility to study the transition between finite and bulk systems.

**Figure 9:**
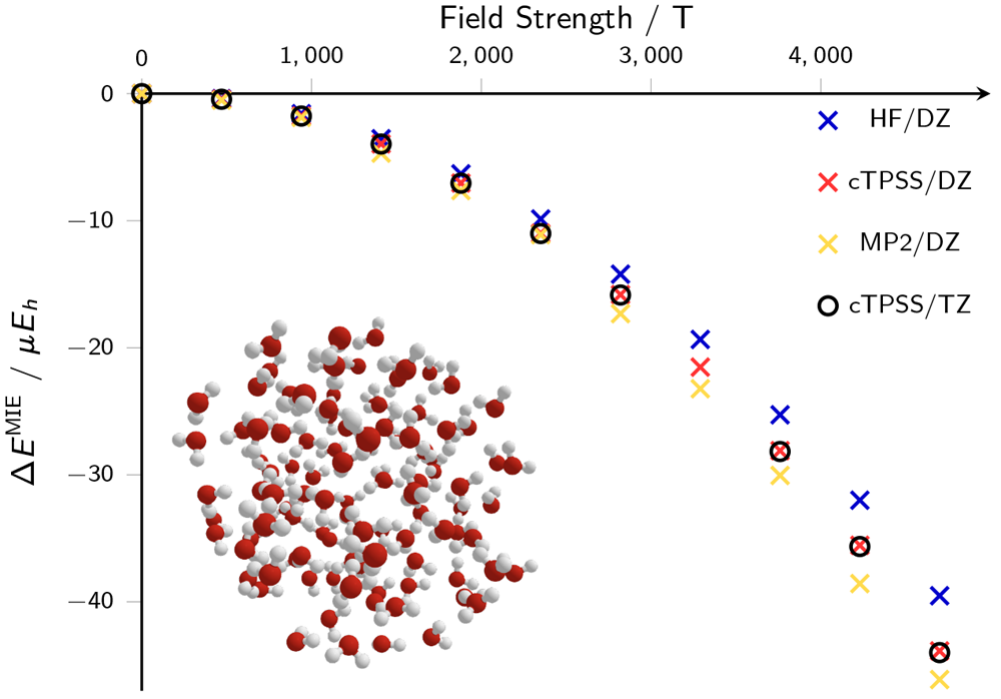
Change in the mean interaction energy for a molecular cluster consisting of 103 water molecules, determined at the HF (blue), DFT/cTPSS (red), and MP2 (yellow) theory levels with the aug-cc-pVDZ basis set and using RI. Equivalent DFT/cTPSS calculations with the aug-cc-pVTZ basis set are also shown as black circles.

A simple measure to compute is the mean interaction energy (MIE) for a system of *n* noncovalently bound molecules,
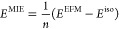
30Figure 9 demonstrates the change in *E*^MIE^ with increasing magnetic field strength applied to a molecular cluster consisting of 103 water molecules, relative to the same quantity in the absence of the external field.

Single-point EFM energy calculations were undertaken in the presence of external magnetic fields ranging from |**B**| = 0.00*B*_0_ to 0.02*B*_0_ with the HF, cTPSS, and MP2 electronic structure methods in the contracted aug-cc-pVDZ basis set and using the RI approximation as described in Section 3. With cTPSS, these calculations were repeated in the larger aug-cc-pVTZ basis set to examine the effects of basis set incompleteness on the results; very small differences in the mean interaction energies were observed relative to the values obtained in the smaller aug-cc-pVDZ basis set, indicating that the smaller basis set is adequate to obtain reasonably converged interaction energies in larger clusters. We note that a conventional calculation with the aug-cc-pVTZ basis set would require an LAO-based calculation with more than 12 000 basis functions and the EFM plays an essential role in making such calculations tractable.

For these larger cluster sizes, it was observed that, in some cases, convergence difficulties in the self-consistent field (SCF) procedure were encountered when employing density-functional methods with the standard embedding potential of eq 8. The origin of these effects was traced to the spurious short-range behavior of this simple dipole model, as discussed in Section 3.2, coupled with delocalization error of conventional density-functional methods leading to electron densities with more pronounced long-range interactions.^130^ To mitigate these SCF convergence problems all cTPSS calculations here include short-range attenuation of the embedding potential based on eq 24 with an attenuation parameter μ = 0.45. This value was chosen pragmatically by reducing μ to the largest value allowing robust convergence, therefore keeping the embedding potential as similar as possible to its unattenuated form. The mean interaction energies yielded by these calculations are plotted as a function of magnetic field strength, relative to the value at zero field, in Figure 9.

It is noteworthy that the value of μ chosen in the manner described above is consistent with the analysis of the absolute errors in the energy discussed in Section 3.2. Furthermore, we have confirmed that the interaction energies are insensitive to the choice of μ. For HF and MP2 no SCF convergence errors were observed, consistent with the localization error of HF, and so the conventional embedding potential of eq 8 was employed directly in these calculations. The change in the interaction energies is similar for each method considered and the cTPSS results fall between HF and MP2, as may be expected, based on experience in the absence of an external field.

It can be seen in Figure 9 that, for all methods, the mean interaction energy becomes more negative with increasing field strength; in other words, the intermolecular interaction becomes stronger as the strength of the magnetic field increases. The same trend is observed for all three methods, with small differences in the absolute value most likely attributable to the differences in the treatment of exchange and correlation effects between methods. Furthermore, as demonstrated for cTPSS, the results appear to be almost independent of basis set size. Therefore, it follows that these results provide at least a qualitative agreement with the experimentally observed changes in the physical properties of water under the influence of a magnetic field.^42−44,47,48,50^ However, it is not possible to infer from these results the underlying mechanism responsible for this effect and further investigation would be required to consider this question further.

As described in Section 4.1, it is important to note that this work does not account for the effects of geometry relaxation as the magnetic field strength changes, which may become significant at higher field strengths. However, at the range of field strengths considered in Figure 9, this effect is expected to be minimal and the change in mean interaction energy for a fixed geometry should remain a good indication of how the system responds in the presence of such magnetic fields.

## 5 Conclusions

In this work, a readily accessible method has been presented for undertaking electronic structure calculations on large weakly bound molecular clusters in the presence of arbitrary strength magnetic fields using any method applicable to simple monomer and dimer calculations. This generalization of the embedded fragment method of ref (55) builds on recent developments in the nonperturbative treatment of magnetic fields in electronic structure calculations while addressing a fundamental limitation of such methods, high computational cost, thus permitting for the first time the effects of magnetic fields on large noncovalently bound molecular clusters to be examined.

It has been shown that the generalized EFM remains accurate in strong magnetic fields, with the level of accuracy remaining similar to that at zero field as the field strength is increased up to |**B**| = 0.3 *B*_0_. Furthermore, it has been shown that the computational advantages of fragmentation-based approaches are maintained in this generalization, with the expected reduction in scaling from , 3 ≤ *x* ≤ 7 (depending on the electronic structure method chosen), to , with respect to system size. The present implementation is also comprehensively parallelized to make full use of modern high-performance compute resources.

This method has been applied to large water clusters to enable the theoretical study of how the intermolecular interactions within the water cluster respond to the presence of an external magnetic field. To address convergence issues sometimes observed for clusters of this size, a simple and inexpensive modification to the dipole embedding potential was developed. Introducing attenuation at short-range, where the representation of the Coulomb potential is least accurate, addresses convergence difficulties arising when monomers are not well separated without compromising the accuracy of the method.

The observed trend in the change of the mean interaction energy with magnetic field strength demonstrated that, as the field strength increases, interactions between the water molecules are strengthened, which is consistent with the results of previous experimental studies.^42,43,47,48,50^ Many of these studies attribute this to a strengthening of the hydrogen bonding network within water. However, the change in charge density with field strength shown in Figure 6 does not reveal an obvious shift in the electron density corresponding to a strengthening of the hydrogen bonds. In addition, the changes in charge density associated with the magnetically enhanced binding are only significant at magnetic field strengths well above that which would be possible in a laboratory setting. Combined with the observations from Figure 6, this increase in interaction strength cannot be confidently attributed to an increase in the hydrogen bonding as often hypothesized from experimental results. This has been further confirmed with analysis of the change in the electron density for larger clusters, which are consistent with the change shown in Figure 6. Because of occlusion effects, however, these are difficult to visualize and therefore images for these cases have not been included here.

In the present work, only relatively weak magnetic field strengths (in atomic units) were considered for the larger clusters, where structural perturbations are expected to be relatively modest. Nonetheless, given the weak nature of the interactions it would be interesting to take into account geometrical relaxation and the implementation of analytic gradients building upon the implementation in ref (28) is currently underway. This initial study has also focused on homogeneous water clusters to investigate the capabilities of the EFM approach in the presence of external magnetic fields. Future work will use this framework for heterogeneous clusters to examine both the influence of external magnetic fields on solute–solvent interactions and as a model for solvation effects in the determination of magnetic response properties.

## Appendix A. RI-MP2/MP3 in Strong Magnetic Fields

In the present work, the RI approximation has been employed to improve the efficiency of calculations for the largest water clusters. In particular, an implementation of MP2 and MP3 using the RI approximation in the basis of LAOs was particularly effective to reduce computational resource requirements. The working equations for RI-MP2 and RI-MP3 in a basis of LAOs for calculations in strong magnetic fields are given here for completeness.

In the RI approximation, two-center charge distributions are expanded in a basis of atom-centered auxiliary functions φ_P_, allowing the four-center electron repulsion integrals to be approximated by the contraction of two and three-center intermediates,

A-1

where the two and three-center intermediates are respectively defined as

A-2

A-3

which is conveniently recast using a further intermediate as

A-4

Standard Gaussian basis functions are used to represent the auxiliary functions, since the use of LAOs for this purpose would result in an unphysical gauge-origin dependence in the charge distribution the auxiliary functions represent. The use of the RI approximation in calculations with LAOs is discussed in refs (28), (56), and (133).

The working equations for evaluating the conventional MP2 correlation energy in a basis of LAOs have been presented elsewhere,^134,135^ and are summarized as

A-5

where *i*, *j* are occupied α orbitals, *a*, *b* are unoccupied α orbitals, and  and  are similar for β orbitals, respectively. Here,  are the excitation amplitudes, given by

A-6

with HF orbital energies ε and two-electron integrals in the molecular orbital (MO) basis that may be expressed in terms of the MO coefficients **c** as

A-7

where the antisymmetrized integrals are given by

A-8

The four-index integral in eq A-7 can be approximated in terms of the MO-transformed intermediates of eq A-4 as

A-9

from which approximations to the amplitudes in eq A-6 can be constructed as

A-10

and with which the MP2 correlation energy of eq A-5 may be rewritten as

A-11

As a result, only the MO transformed three-index intermediates of eq A-4 are required in the RI approximation. This reduces both the computational cost and memory requirements of the integral transformation step and subsequent MP2 correlation energy calculation.

The working equations for the MP3 energy are significantly longer than those of the MP2 energy, since the higher-order in perturbation theory introduces many additional terms.^136,137^ In its conventional form, the MP3 energy may be written in a similar way to eq A-5 as

A-12

where  are the intermediates defined as

A-13

and, similarly,  terms are defined as

A-14

These may be approximated by three-center integrals in the same way as the MP2 amplitudes are in eq A-10, respectively constructed as

A-15

and

A-16

## Supporting Information Available

Conventional and EFM energies for the water trimer used to calculate the errors in Table 1; conventional and EFM energies for the series of water clusters used to calculate the errors in Figure 4; geometry of the 103 molecule water cluster discussed in Section 4.3 and shown in Figure 9 (PDF)

## References

[ref1] Xantheas S. S.; Dunning T. H. *ab initio* studies of cyclic water clusters (H_2_O)_*n*_, *n* = 1–6. I. Optimal structures and vibrational spectra. J. Chem. Phys. 1993, 99, 8774–8792. 10.1063/1.465599.

[ref2] Xantheas S. S. *ab initio* studies of cyclic water clusters (H_2_O)_*n*_, n = 1–6. II. Analysis of many-body interactions. J. Chem. Phys. 1994, 100, 7523–7534. 10.1063/1.466846.

[ref3] Burnham C. J.; Xantheas S. S. Development of transferable interaction models for water. IV. A flexible, all-atom polarizable potential (TTM2-F) based on geometry dependent charges derived from an *ab initio* monomer dipole moment surface. J. Chem. Phys. 2002, 116, 5115 10.1063/1.1447904.

[ref4] Xantheas S. S.; Burnham C. J.; Harrison R. J. Development of transferable interaction models for water. II. Accurate energetics of the first few water clusters from first principles. J. Chem. Phys. 2002, 116, 1493–1499. 10.1063/1.1423941.

[ref5] Kohn W. Density functional theory for systems of very many atoms. Int. J. Quantum Chem. 1995, 56, 229–232. 10.1002/qua.560560407.

[ref6] Head-Gordon M. Quantum Chemistry and Molecular Processes. J. Phys. Chem. 1996, 100, 13213–13225. 10.1021/jp953665+.

[ref7] Almlöf J.; Fægri K.; Korsell K. Principles for a direct SCF approach LCAO-MO *ab initio* calculations. J. Comput. Chem. 1982, 3, 385–399. 10.1002/jcc.540030314.

[ref8] Reine S.; Tellgren E.; Krapp A.; Kjærgaard T.; Helgaker T.; Jansik B.; Høst S.; Salek P. Variational and robust density fitting of four-center two-electron integrals in local metrics. J. Chem. Phys. 2008, 129, 104101 10.1063/1.2956507. 19044902

[ref9] Neese F.; Wennmohs F.; Hansen A.; Becker U. Efficient, approximate and parallel Hartree–Fock and hybrid DFT calculations. A ‘chain-of-spheres’ algorithm for the Hartree–Fock exchange. Chem. Phys. 2009, 356, 98–109. 10.1016/j.chemphys.2008.10.036.

[ref10] Henderson T. M. Embedding wave function theory in density functional theory. J. Chem. Phys. 2006, 125, 014105 10.1063/1.2209688. 16863285

[ref11] Dresselhaus T.; Neugebauer J. Part and whole in wavefunction/DFT embedding. Theor. Chem. Acc. 2015, 134, 10.1007/s00214-015-1697-4.

[ref12] Lee S. J. R.; Welborn M.; Manby F. R.; Miller T. F. Projection-Based Wavefunction-in-DFT Embedding. Acc. Chem. Res. 2019, 52, 1359–1368. 10.1021/acs.accounts.8b00672. 30969117

[ref13] Gordon M. S.; Fedorov D. G.; Pruitt S. R.; Slipchenko L. V. Fragmentation Methods: A Route to Accurate Calculations on Large Systems. Chem. Rev. 2012, 112, 632–672. 10.1021/cr200093j. 21866983

[ref14] Cisneros G. A.; Wikfeldt K. T.; Ojamäe L.; Lu J.; Xu Y.; Torabifard H.; Bartók A. P.; Csányi G.; Molinero V.; Paesani F. Modeling Molecular Interactions in Water: From Pairwise to Many-Body Potential Energy Functions. Chem. Rev. 2016, 116, 7501–7528. 10.1021/acs.chemrev.5b00644. 27186804 PMC5450669

[ref15] Richard R. M.; Lao K. U.; Herbert J. M. Aiming for Benchmark Accuracy with the Many-Body Expansion. Acc. Chem. Res. 2014, 47, 2828–2836. 10.1021/ar500119q. 24883986

[ref16] Tellgren E. I.; Soncini A.; Helgaker T. Nonperturbative *ab initio* calculations in strong magnetic fields using London orbitals. J. Chem. Phys. 2008, 129, 154114 10.1063/1.2996525. 19045183

[ref17] Tellgren E. I.; Helgaker T.; Soncini A. Non-perturbative magnetic phenomena in closed-shell paramagnetic molecules. Phys. Chem. Chem. Phys. 2009, 11, 5489 10.1039/b822262b. 19551219

[ref18] Tellgren E. I.; Kvaal S.; Sagvolden E.; Ekström U.; Teale A. M.; Helgaker T. Choice of basic variables in current-density-functional theory. Phys. Rev. A 2012, 86, 062506 10.1103/PhysRevA.86.062506.

[ref19] Tellgren E. I.; Teale A. M.; Furness J. W.; Lange K. K.; Ekström U.; Helgaker T. Non-perturbative calculation of molecular magnetic properties within current-density functional theory. J. Chem. Phys. 2014, 140, 034101 10.1063/1.4861427. 25669357

[ref20] Stopkowicz S.; Gauss J.; Lange K. K.; Tellgren E. I.; Helgaker T. Coupled-cluster theory for atoms and molecules in strong magnetic fields. J. Chem. Phys. 2015, 143, 074110 10.1063/1.4928056. 26298118

[ref21] Sun S.; Williams-Young D. B.; Stetina T. F.; Li X. Generalized Hartree–Fock with Nonperturbative Treatment of Strong Magnetic Fields: Application to Molecular Spin Phase Transitions. J. Chem. Theory Comput. 2019, 15, 348–356. 10.1021/acs.jctc.8b01140. 30485745

[ref22] Sun S.; Williams-Young D.; Li X. An *ab Initio* Linear Response Method for Computing Magnetic Circular Dichroism Spectra with Nonperturbative Treatment of Magnetic Field. J. Chem. Theory Comput. 2019, 15, 3162–3169. 10.1021/acs.jctc.9b00095. 30933558

[ref23] Furness J. W.; Verbeke J.; Tellgren E. I.; Stopkowicz S.; Ekström U.; Helgaker T.; Teale A. M. Current Density Functional Theory Using Meta-Generalized Gradient Exchange-Correlation Functionals. J. Chem. Theory Comput. 2015, 11, 4169–4181. 10.1021/acs.jctc.5b00535. 26575912

[ref24] Hampe F.; Stopkowicz S. Equation-of-motion coupled-cluster methods for atoms and molecules in strong magnetic fields. J. Chem. Phys. 2017, 146, 154105 10.1063/1.4979624. 28433009

[ref25] Reimann S.; Borgoo A.; Austad J.; Tellgren E. I.; Teale A. M.; Helgaker T.; Stopkowicz S. Kohn–Sham energy decomposition for molecules in a magnetic field. Mol. Phys. 2019, 117, 97–109. 10.1080/00268976.2018.1495849.

[ref26] Wibowo M.; Irons T. J. P.; Teale A. M. Modeling Ultrafast Electron Dynamics in Strong Magnetic Fields Using Real-Time Time-Dependent Electronic Structure Methods. J. Chem. Theory Comput. 2021, 17, 2137–2165. 10.1021/acs.jctc.0c01269. 33724806 PMC8047917

[ref27] Irons T. J. P.; Zemen J.; Teale A. M. Efficient Calculation of Molecular Integrals over London Atomic Orbitals. J. Chem. Theory Comput. 2017, 13, 3636–3649. 10.1021/acs.jctc.7b00540. 28692291

[ref28] Irons T. J. P.; David G.; Teale A. M. Optimizing Molecular Geometries in Strong Magnetic Fields. J. Chem. Theory Comput. 2021, 17, 2166–2185. 10.1021/acs.jctc.0c01297. 33724812 PMC8047810

[ref29] David G.; Irons T. J. P.; Fouda A. E. A.; Furness J. W.; Teale A. M. Self-Consistent Field Methods for Excited States in Strong Magnetic Fields: a Comparison between Energy- and Variance-Based Approaches. J. Chem. Theory Comput. 2021, 17, 5492–5508. 10.1021/acs.jctc.1c00236. 34517708

[ref30] Pemberton M. J.; Irons T. J. P.; Helgaker T.; Teale A. M. Revealing the exotic structure of molecules in strong magnetic fields. J. Chem. Phys. 2022, 156, 204113 10.1063/5.0092520. 35649858

[ref31] Angel J. R. P.; Landstreet J. D. A Determination by the Zeeman Effect of the Magnetic Field Strength in the White Dwarf G99–37. Astrophys. J. 1974, 191, 457 10.1086/152984.

[ref32] Angel J. R. P. Magnetism in white dwarfs. Astrophys. J. 1977, 216, 1 10.1086/155436.

[ref33] Ivanov M. V. Hartree-Fock calculation of the 1*s*^2^ 2*s*^2^ state of the Be atom in external magnetic fields from γ = 0 up to γ = 1000. Phys. Rev. A 1998, 239, 72–80. 10.1016/S0375-9601(97)00937-7.

[ref34] Ivanov M. V.; Schmelcher P. Ground state of the lithium atom in strong magnetic fields. Phys. Rev. A 1998, 57, 3793–3800. 10.1103/PhysRevA.57.3793.

[ref35] Ivanov M. V.; Schmelcher P. Ground state of the carbon atom in strong magnetic fields. Phys. Rev. A 1999, 60, 3558–3568. 10.1103/PhysRevA.60.3558.

[ref36] LONDON A. quantum chemistry program for plane–wave/GTO hybrid basis sets and finite magnetic field calculations. londonprogram.org..

[ref37] BAGEL, Brilliantly Advanced General Electronic-structure Library. nubakery.org, Published under the GNU General Public License.

[ref38] TURBOMOLE V6.2. turbomole.com, 2010; A development of University of Karlsruhe and Forschungszentrum Karlsruhe GmbH, 1989–2007, TURBOMOLE GmbH, since 2007.

[ref39] QUEST A. rapid development platform for Quantum Electronic Structure Techniques. quest.codes, 2017.

[ref40] Williams-Young D. B.; Petrone A.; Sun S.; Stetina T. F.; Lestrange P.; Hoyer C. E.; Nascimento D. R.; Koulias L.; Wildman A.; Kasper J.; Goings J. J.; Ding F.; DePrince A. E.; Valeev E. F.; Li X. The Chronus Quantum software package. WIREs Comput. Mol. Sci. 2020, 10, 10.1002/wcms.1436.

[ref41] Lange K. K.; Tellgren E. I.; Hoffmann M. R.; Helgaker T. A Paramagnetic Bonding Mechanism for Diatomics in Strong Magnetic Fields. Science 2012, 337, 327–331. 10.1126/science.1219703. 22822146

[ref42] Iwasaka M.; Ueno S. Structure of water molecules under 14 T magnetic field. J. Appl. Phys. 1998, 83, 6459–6461. 10.1063/1.367737.

[ref43] Hosoda H.; Mori H.; Sogoshi N.; Nagasawa A.; Nakabayashi S. Refractive Indices of Water and Aqueous Electrolyte Solutions under High Magnetic Fields. J. Phys. Chem. A 2004, 108, 1461–1464. 10.1021/jp0310145.

[ref44] Inaba H.; Saitou T.; Tozaki K.-i.; Hayashi H. Effect of the magnetic field on the melting transition of H_2_O and D_2_O measured by a high resolution and supersensitive differential scanning calorimeter. J. Appl. Phys. 2004, 96, 6127–6132. 10.1063/1.1803922.

[ref45] Amiri M. C.; Dadkhah A. A. On reduction in the surface tension of water due to magnetic treatment. Colloids Surf., A 2006, 278, 252–255. 10.1016/j.colsurfa.2005.12.046.

[ref46] Holysz L.; Szczes A.; Chibowski E. Effects of a static magnetic field on water and electrolyte solutions. J. Colloid Interface Sci. 2007, 316, 996–1002. 10.1016/j.jcis.2007.08.026. 17897662

[ref47] Toledo E. J. L.; Ramalho T. C.; Magriotis Z. M. Influence of magnetic field on physical-chemical properties of the liquid water: Insights from experimental and theoretical models. J. Mol. Struct. 2008, 888, 409–415. 10.1016/j.molstruc.2008.01.010.

[ref48] Cai R.; Yang H.; He J.; Zhu W. The effects of magnetic fields on water molecular hydrogen bonds. J. Mol. Struct. 2009, 938, 15–19. 10.1016/j.molstruc.2009.08.037.

[ref49] Iino M.; Fujimura Y. Surface tension of heavy water under high magnetic fields. Appl. Phys. Lett. 2009, 94, 261902 10.1063/1.3167767.

[ref50] Wang Y.; Zhang B.; Gong Z.; Gao K.; Ou Y.; Zhang J. The effect of a static magnetic field on the hydrogen bonding in water using frictional experiments. J. Mol. Struct. 2013, 1052, 102–104. 10.1016/j.molstruc.2013.08.021.

[ref51] Wang Y.; Wei H.; Li Z. Effect of magnetic field on the physical properties of water. Results Phys. 2018, 8, 262–267. 10.1016/j.rinp.2017.12.022.

[ref52] Kobe S.; Dražić G.; McGuiness P. J.; Stražišar J. The influence of the magnetic field on the crystallisation form of calcium carbonate and the testing of a magnetic water-treatment device. J. Magn. Magn. Mater. 2001, 236, 71–76. 10.1016/S0304-8853(01)00432-2.

[ref53] Liu B.; Gao B.; Xu X.; Hong W.; Yue Q.; Wang Y.; Su Y. The combined use of magnetic field and iron-based complex in advanced treatment of pulp and paper wastewater. Chem. Eng. J. 2011, 178, 232–238. 10.1016/j.cej.2011.10.058.

[ref54] Wei H.; Wang Y.; Luo J. Influence of magnetic water on early-age shrinkage cracking of concrete. Constr Build Mater. 2017, 147, 91–100. 10.1016/j.conbuildmat.2017.04.140.

[ref55] Hirata S.; Valiev M.; Dupuis M.; Xantheas S. S.; Sugiki S.; Sekino H. Fast electron correlation methods for molecular clusters in the ground and excited states. Mol. Phys. 2005, 103, 2255–2265. 10.1080/00268970500083788.

[ref56] Pausch A.; Klopper W. Efficient evaluation of three-centre two-electron integrals over London orbitals. Mol. Phys. 2020, 118, e1736675 10.1080/00268976.2020.1736675.

[ref57] Blaschke S.; Stopkowicz S. Cholesky decomposition of complex two-electron integrals over GIAOs: Efficient MP2 computations for large molecules in strong magnetic fields. J. Chem. Phys. 2022, 156, 044115 10.1063/5.0076588. 35105060

[ref58] Hankins D.; Moskowitz J. W.; Stillinger F. H. Water Molecule Interactions. J. Chem. Phys. 1970, 53, 4544–4554. 10.1063/1.1673986.

[ref59] Elrod M. J.; Saykally R. J. Many-Body Effects in Intermolecular Forces. Chem. Rev. 1994, 94, 1975–1997. 10.1021/cr00031a010. 11539597

[ref60] Kulkarni A. D.; Ganesh V.; Gadre S. R. Many-body interaction analysis: Algorithm development and application to large molecular clusters. J. Chem. Phys. 2004, 121, 5043–5050. 10.1063/1.1780156. 15352794

[ref61] Pruitt S. R.; Bertoni C.; Brorsen K. R.; Gordon M. S. Efficient and Accurate Fragmentation Methods. Acc. Chem. Res. 2014, 47, 2786–2794. 10.1021/ar500097m. 24810424

[ref62] Collins M. A.; Bettens R. P. A. Energy-Based Molecular Fragmentation Methods. Chem. Rev. 2015, 115, 5607–5642. 10.1021/cr500455b. 25843427

[ref63] Liu K.-Y.; Herbert J. M. Understanding the many-body expansion for large systems. III. Critical role of four-body terms, counterpoise corrections, and cutoffs. J. Chem. Phys. 2017, 147, 161729 10.1063/1.4986110. 29096456

[ref64] Saha S.; Vivek M. R.; Sastry G. N. On the origin of spurious errors in many-body expansion for water cluster. J. Chem. Sci. 2017, 129, 1053–1060. 10.1007/s12039-017-1303-5.

[ref65] Cui J.; Liu H.; Jordan K. D. Theoretical Characterization of the (H_2_O)_21_ Cluster: Application of an *n*-body Decomposition Procedure. J. Phys. Chem. B 2006, 110, 18872–18878. 10.1021/jp056416m. 16986878

[ref66] Chen Y.; Li H. Intermolecular Interaction in Water Hexamer. J. Phys. Chem. A 2010, 114, 11719–11724. 10.1021/jp104822e. 20932059

[ref67] Herbert J. M. Fantasy versus reality in fragment-based quantum chemistry. J. Chem. Phys. 2019, 151, 170901 10.1063/1.5126216. 31703524

[ref68] Lao K. U.; Liu K.-Y.; Richard R. M.; Herbert J. M. Understanding the many-body expansion for large systems. II. Accuracy considerations. J. Chem. Phys. 2016, 144, 164105 10.1063/1.4947087. 27131529

[ref69] Richard R. M.; Lao K. U.; Herbert J. M. Understanding the many-body expansion for large systems. I. Precision considerations. J. Chem. Phys. 2014, 141, 014108 10.1063/1.4885846. 25005278

[ref70] Axilrod B. M.; Teller E. Interaction of the van der Waals Type Between Three Atoms. J. Chem. Phys. 1943, 11, 299–300. 10.1063/1.1723844.

[ref71] Muto Y. Force between nonpolar molecules. Proc. Phys.-Math. Soc. Jpn. 1943, 17, 629–631.

[ref72] Clementi E.; Kołos W.; Lie G. C.; Ranghino G. Nonadditivity of interaction in water trimers. Int. J. Quantum Chem. 1980, 17, 377–398. 10.1002/qua.560170302.

[ref73] Dahlke E. E.; Truhlar D. G. Electrostatically Embedded Many-Body Expansion for Large Systems, with Applications to Water Clusters. J. Chem. Theory Comput. 2007, 3, 46–53. 10.1021/ct600253j. 26627150

[ref74] Cloizeaux J. D. Energy Bands and Projection Operators in a Crystal: Analytic and Asymptotic Properties. Phys. Rev. 1964, 135, A685–A697. 10.1103/PhysRev.135.A685.

[ref75] Piela L.; André J.-M.; Fripiat J. G.; Delhalle J. On the behaviour of exchange in restricted hartree-fock-roothaan calculations for periodic polymers. Chem. Phys. Lett. 1981, 77, 143–150. 10.1016/0009-2614(81)85617-5.

[ref76] Monkhorst H. J.; Kertesz M. Exact-exchange asymptotics in polymer Hartree-Fock calculations. Phys. Rev. B 1981, 24, 3015–3024. 10.1103/PhysRevB.24.3015.

[ref77] Gillan M. J.; Alfè D.; Bygrave P. J.; Taylor C. R.; Manby F. R. Energy benchmarks for water clusters and ice structures from an embedded many-body expansion. J. Chem. Phys. 2013, 139, 114101 10.1063/1.4820906. 24070273

[ref78] Heßelmann A. Correlation effects and many-body interactions in water clusters. Beilstein J. Org. Chem. 2018, 14, 979–991. 10.3762/bjoc.14.83. 29977369 PMC6009095

[ref79] Keesom W. H. On the second virial coefficient for di-atomic gases. K. Ned. Akad. Wet., Proc., Ser. B: Phys. Sci. 1912, 15, 417–431.

[ref80] London F. Théorie quantique des courants interatomiques dans les combinaisons aromatiques. J. Phys. Radium 1937, 8, 397–409. 10.1051/jphysrad:01937008010039700.

[ref81] Debye P. The van der Waals cohesion forces. Phys. Z. 1920, 21, 178–187.

[ref82] Debye P. Molecular forces and their electric explanation. Phys. Z. 1921, 22, 302–308.

[ref83] London F. Zur Theorie und Systematik der Molekularkräfte. Z. Phys. 1930, 63, 245–279. 10.1007/BF01421741.

[ref84] Chen W.; Gordon M. S. Energy Decomposition Analyses for Many-Body Interaction and Applications to Water Complexes. J. Phys. Chem. 1996, 100, 14316–14328. 10.1021/jp960694r.

[ref85] Raghavachari K.; Saha A. Accurate Composite and Fragment-Based Quantum Chemical Models for Large Molecules. Chem. Rev. 2015, 115, 5643–5677. 10.1021/cr500606e. 25849163

[ref86] Dahlke E. E.; Truhlar D. G. Assessment of the Pairwise Additive Approximation and Evaluation of Many-Body Terms for Water Clusters. J. Phys. Chem. B 2006, 110, 10595–10601. 10.1021/jp061039e. 16771303

[ref87] Dahlke E. E.; Truhlar D. G. Electrostatically Embedded Many-Body Expansion for Simulations. J. Chem. Theory Comput. 2008, 4, 1–6. 10.1021/ct700223r. 26619974

[ref88] Dahlke E. E.; Truhlar D. G. Electrostatically Embedded Many-Body Correlation Energy, with Applications to the Calculation of Accurate Second-Order Møller-Plesset Perturbation Theory Energies for Large Water Clusters. J. Chem. Theory Comput. 2007, 3, 1342–1348. 10.1021/ct700057x. 26633207

[ref89] Kitaura K.; Ikeo E.; Asada T.; Nakano T.; Uebayasi M. Fragment molecular orbital method: an approximate computational method for large molecules. Chem. Phys. Lett. 1999, 313, 701–706. 10.1016/S0009-2614(99)00874-X.

[ref90] Fedorov D. G.; Kitaura K. Extending the Power of Quantum Chemistry to Large Systems with the Fragment Molecular Orbital Method. J. Phys. Chem. A 2007, 111, 6904–6914. 10.1021/jp0716740. 17511437

[ref91] Fedorov D. G.; Nagata T.; Kitaura K. Exploring chemistry with the fragment molecular orbital method. Phys. Chem. Chem. Phys. 2012, 14, 7562 10.1039/c2cp23784a. 22410762

[ref92] Fedorov D. G.; Kitaura K. On the accuracy of the 3-body fragment molecular orbital method (FMO) applied to density functional theory. Chem. Phys. Lett. 2004, 389, 129–134. 10.1016/j.cplett.2004.03.072.

[ref93] Nakano T.; Mochizuki Y.; Yamashita K.; Watanabe C.; Fukuzawa K.; Segawa K.; Okiyama Y.; Tsukamoto T.; Tanaka S. Development of the four-body corrected fragment molecular orbital (FMO4) method. Chem. Phys. Lett. 2012, 523, 128–133. 10.1016/j.cplett.2011.12.004.

[ref94] Fedorov D. G.; Kitaura K. Coupled-cluster theory based upon the fragment molecular-orbital method. J. Chem. Phys. 2005, 123, 134103 10.1063/1.2007588. 16223271

[ref95] Nakata H.; Fedorov D. G.; Yokojima S.; Kitaura K.; Sakurai M.; Nakamura S. Unrestricted density functional theory based on the fragment molecular orbital method for the ground and excited state calculations of large systems. J. Chem. Phys. 2014, 140, 144101 10.1063/1.4870261. 24735282

[ref96] Jansen H. B.; Ros P. Non-empirical molecular orbital calculations on the protonation of carbon monoxide. Chem. Phys. Lett. 1969, 3, 140–143. 10.1016/0009-2614(69)80118-1.

[ref97] Liu B.; McLean A. D. Accurate calculation of the attractive interaction of two ground state helium atoms. J. Chem. Phys. 1973, 59, 4557–4558. 10.1063/1.1680654.

[ref98] Simon S.; Duran M.; Dannenberg J. J. How does basis set superposition error change the potential surfaces for hydrogen-bonded dimers?. J. Chem. Phys. 1996, 105, 11024–11031. 10.1063/1.472902.

[ref99] Salvador P.; Szczȩśniak M. M. Counterpoise-corrected geometries and harmonic frequencies of N-body clusters: Application to (HF)_*n*_ (*n* = 3,4). J. Chem. Phys. 2003, 118, 537–549. 10.1063/1.1527011.

[ref100] van Duijneveldt F. B.; van Duijneveldt-van de Rijdt J. G. C. M.; van Lenthe J. H. State of the Art in Counterpoise Theory. Chem. Rev. 1994, 94, 1873–1885. 10.1021/cr00031a007.

[ref101] Boys S. F.; Bernardi F. The calculation of small molecular interactions by the differences of separate total energies. Some procedures with reduced errors. Mol. Phys. 1970, 19, 553–566. 10.1080/00268977000101561.

[ref102] Wells B. H.; Wilson S. van der Waals interaction potentials: Many-body basis set superposition effects. Chem. Phys. Lett. 1983, 101, 429–434. 10.1016/0009-2614(83)87508-3.

[ref103] Turi L.; Dannenberg J. J. Correcting for basis set superposition error in aggregates containing more than two molecules: ambiguities in the calculation of the counterpoise correction. J. Phys. Chem. 1993, 97, 2488–2490. 10.1021/j100113a002.

[ref104] Valiron P.; Mayer I. Hierarchy of counterpoise corrections for N-body clusters: generalization of the Boys-Bernardi scheme. Chem. Phys. Lett. 1997, 275, 46–55. 10.1016/S0009-2614(97)00689-1.

[ref105] Ouyang J. F.; Bettens R. P. A. Many-Body Basis Set Superposition Effect. J. Chem. Theory Comput. 2015, 11, 5132–5143. 10.1021/acs.jctc.5b00343. 26574311

[ref106] Kamiya M.; Hirata S.; Valiev M. Fast electron correlation methods for molecular clusters without basis set superposition errors. J. Chem. Phys. 2008, 128, 074103 10.1063/1.2828517. 18298136

[ref107] Ditchfield R. Self-consistent perturbation theory of diamagnetism. Mol. Phys. 1974, 27, 789–807. 10.1080/00268977400100711.

[ref108] Hohenberg P.; Kohn W. Inhomogeneous Electron Gas. Phys. Rev. 1964, 136, B864–B871. 10.1103/PhysRev.136.B864.

[ref109] Kohn W.; Sham L. J. Self-Consistent Equations Including Exchange and Correlation Effects. Phys. Rev. 1965, 140, A1133–A1138. 10.1103/PhysRev.140.A1133.

[ref110] Grayce C. J.; Harris R. A. Magnetic-field density-functional theory. Phys. Rev. A 1994, 50, 3089–3095. 10.1103/PhysRevA.50.3089. 9911249

[ref111] Salsbury F. R.; Harris R. A. The current in magnetic field density functional theory and its application to the chemical shielding and magnetic susceptibility. J. Chem. Phys. 1997, 107, 7350–7359. 10.1063/1.475165.

[ref112] Vignale G.; Rasolt M. Density-functional theory in strong magnetic fields. Phys. Rev. Lett. 1987, 59, 2360–2363. 10.1103/PhysRevLett.59.2360. 10035523

[ref113] Vignale G.; Rasolt M. Current- and spin-density-functional theory for inhomogeneous electronic systems in strong magnetic fields. Phys. Rev. B 1988, 37, 10685–10696. 10.1103/PhysRevB.37.10685. 9944522

[ref114] Lieb E. H. Density functionals for Coulomb systems. Int. J. Quantum Chem. 1983, 24, 243–277. 10.1002/qua.560240302.

[ref115] Kvaal S.; Laestadius A.; Tellgren E.; Helgaker T. Lower Semicontinuity of the Universal Functional in Paramagnetic Current–Density Functional Theory. J. Phys. Chem. Lett. 2021, 12, 1421–1425. 10.1021/acs.jpclett.0c03422. 33522817 PMC7883387

[ref116] Dobson J. F. Spin-density functionals for the electron correlation energy with automatic freedom from orbital self-interaction. J. Phys.: Condens. Matter 1992, 4, 7877–7890. 10.1088/0953-8984/4/39/003.

[ref117] Dobson J. F. Alternative expressions for the Fermi hole curvature. J. Chem. Phys. 1993, 98, 8870–8872. 10.1063/1.464444.

[ref118] Becke A. D. Current-density dependent exchange-correlation functionals. Can. J. Chem. 1996, 74, 995–997. 10.1139/v96-110.

[ref119] Tao J.; Perdew J. P.; Staroverov V. N.; Scuseria G. E. Climbing the Density Functional Ladder: Nonempirical Meta–Generalized Gradient Approximation Designed for Molecules and Solids. Phys. Rev. Lett. 2003, 91, 10.1103/PhysRevLett.91.146401. 14611541

[ref120] Tao J. Explicit inclusion of paramagnetic current density in the exchange-correlation functionals of current-density functional theory. Phys. Rev. B 2005, 71, 71 10.1103/PhysRevB.71.205107.

[ref121] Dalcin L.; Fang Y.-L. L. mpi4py: Status Update After 12 Years of Development. Comput. Sci. Eng. 2021, 23, 47–54. 10.1109/MCSE.2021.3083216.33967632

[ref122] Dunning T. H. Gaussian basis sets for use in correlated molecular calculations. I. The atoms boron through neon and hydrogen. J. Chem. Phys. 1989, 90, 1007 10.1063/1.456153.

[ref123] Kendall R. A.; Dunning T. H.; Harrison R. J. Electron affinities of the first-row atoms revisited. Systematic basis sets and wave functions. J. Chem. Phys. 1992, 96, 6796–6806. 10.1063/1.462569.

[ref124] Woon D. E.; Dunning T. H. Gaussian basis sets for use in correlated molecular calculations. III. The atoms aluminum through argon. J. Chem. Phys. 1993, 98, 1358–1371. 10.1063/1.464303.

[ref125] Stoychev G. L.; Auer A. A.; Neese F. Automatic Generation of Auxiliary Basis Sets. J. Chem. Theory Comput. 2017, 13, 554–562. 10.1021/acs.jctc.6b01041. 28005364

[ref126] Bates D. M.; Smith J. R.; Tschumper G. S. Efficient and Accurate Methods for the Geometry Optimization of Water Clusters: Application of Analytic Gradients for the Two-Body:Many-Body QM:QM Fragmentation Method to (H_2_O)_*n*_, *n* = 3 – 10. J. Chem. Theory Comput. 2011, 7, 2753–2760. 10.1021/ct200176t. 26605466

[ref127] Jorgensen W. L.; Chandrasekhar J.; Madura J. D.; Impey R. W.; Klein M. L. Comparison of simple potential functions for simulating liquid water. J. Chem. Phys. 1983, 79, 926–935. 10.1063/1.445869.

[ref128] Neria E.; Fischer S.; Karplus M. Simulation of activation free energies in molecular systems. J. Chem. Phys. 1996, 105, 1902–1921. 10.1063/1.472061.

[ref129] Todorov I. T.; Smith W.; Trachenko K.; Dove M. T. DL_POLY_3: new dimensions in molecular dynamics simulations via massive parallelism. J. Mater. Chem. 2006, 16, 1911 10.1039/b517931a.

[ref130] Cohen A. J.; Mori-Sánchez P.; Yang W. Insights into Current Limitations of Density Functional Theory. Science 2008, 321, 792–794. 10.1126/science.1158722. 18687952

[ref131] Stone A. J. Electrostatic Damping Functions and the Penetration Energy. J. Phys. Chem. A 2011, 115, 7017–7027. 10.1021/jp112251z. 21619003

[ref132] Ishikawa T. *ab initio* quantum chemical calculation of electron density, electrostatic potential, and electric field of biomolecule based on fragment molecular orbital method. Int. J. Quantum Chem. 2018, 118, e25535 10.1002/qua.25535.

[ref133] Reynolds R. D.; Shiozaki T. Fully relativistic self-consistent field under a magnetic field. Phys. Chem. Chem. Phys. 2015, 17, 14280–14283. 10.1039/C4CP04027A. 25310527

[ref134] Gauss J. Calculation of NMR chemical shifts at second-order many-body perturbation theory using gauge-including atomic orbitals. Chem. Phys. Lett. 1992, 191, 614–620. 10.1016/0009-2614(92)85598-5.

[ref135] Gauss J. Effects of electron correlation in the calculation of nuclear magnetic resonance chemical shifts. J. Chem. Phys. 1993, 99, 3629–3643. 10.1063/1.466161.

[ref136] Bartlett R. J.; Silver D. M. Many-body perturbation theory applied to electron pair correlation energies. I. Closed-shell first-row diatomic hydrides. J. Chem. Phys. 1975, 62, 3258–3268. 10.1063/1.430878.

[ref137] Pople J. A.; Binkley J. S.; Seeger R. Theoretical models incorporating electron correlation. Int. J. Quantum Chem. 1976, 10, 1–19. 10.1002/qua.560100802.

