# Supplementary material for: An Embedded Fragment Method for Molecules in Strong Magnetic Fields
Source: J Chem Theory Comput. 2022 Nov 22;18(12):7412–27. doi: 10.1021/acs.jctc.2c00865 (PMC9753591; doi:10.1021/acs.jctc.2c00865)
Supplement: Supplementary file 1 — ct2c00865_si_001.pdf [file ct2c00865_si_001.pdf]

# Supporting Information

# An Embedded Fragment Method for Molecules in Strong Magnetic Fields

Benjamin T. Speake,[1,a)] Tom J. P. Irons,[1] Meilani Wibowo,[1] Andrew G. Johnson,[1] Grégoire David,[1,2] and Andrew M. Teale[1,3]
[1)] *School of Chemistry, University of Nottingham, University Park, Nottingham, NG7 2RD, UK*
[2)] *Univ Rennes, CNRS, ISCR (Institut des Sciences Chimiques de Rennes)-UMR 6226, F-35000 Rennes, France*
[3)] *Hylleraas Centre for Quantum Molecular Sciences, Department of Chemistry, University of Oslo, P.O. Box 1033 Blindern, N-0315 Oslo, Norway*

(Dated: 20 October 2022)

## I. WATER TRIMER ENERGIES AND ERROR ANALYSIS

TABLE S1: Energies of the water trimer calculated conventionally ($E^{\mathrm{conv}}$), using EFM ($E^{\mathrm{EFM}}$) and as the sum of isolated monomers ($E^{\mathrm{iso}}$), showing the errors in the EFM energies $\delta E$ (excluding the BSSE correction $\delta E^{\mathrm{BSSE}}$) in both $E_h$ and kcalmol$^{-1}$ for a range of electronic structure methods and at increasing magnetic field strengths.

| Method | $\|\mathbf{B}\|$ / $B_0$ | $E^{\mathrm{conv}}$ / $E_h$ | $E^{\mathrm{EFM}}$ / $E_h$ | $\delta E^{\mathrm{BSSE}}$ / $E_h$ | $E^{\mathrm{iso}}$ / $E_h$ | $\delta E$ / $E_h$ | $\delta E$ / kcalmol$^{-1}$ |
|---|---|---|---|---|---|---|---|
| HF | 0.0 | -228.14413040 | -228.14289677 | 0.00146109 | -228.12534048 | -0.00022746 | -0.14 |
| | 0.1 | -228.10169405 | -228.10059461 | 0.00129339 | -228.08150417 | -0.00019395 | -0.12 |
| | 0.2 | -227.97601457 | -227.97495664 | 0.00135645 | -227.95265543 | -0.00029852 | -0.19 |
| | 0.3 | -227.77340786 | -227.77182801 | 0.00196684 | -227.74769980 | -0.00038699 | -0.24 |
| | 0.4 | -227.50860790 | -227.50634420 | 0.00309694 | -227.48463585 | -0.00083324 | -0.52 |
| BLYP | 0.0 | -229.29500794 | -229.29380623 | 0.00209794 | -229.27554724 | -0.00089623 | -0.56 |
| | 0.1 | -229.25179310 | -229.25068142 | 0.00190049 | -229.23034944 | -0.00078881 | -0.49 |
| | 0.2 | -229.12431369 | -229.12324323 | 0.00197684 | -229.09853592 | -0.00090638 | -0.57 |
| | 0.3 | -228.92155928 | -228.91981596 | 0.00272320 | -228.89273230 | -0.00097988 | -0.61 |
| | 0.4 | -228.66526554 | -228.66297562 | 0.00453560 | -228.63828724 | -0.00224568 | -1.41 |
| TPSS | 0.0 | -229.35588658 | -229.35499104 | 0.00182358 | -229.33498799 | -0.00092804 | -0.58 |
| | 0.1 | -229.31307996 | -229.31228907 | 0.00164555 | -229.29044869 | -0.00085466 | -0.54 |
| | 0.2 | -229.18679413 | -229.18605998 | 0.00171028 | -229.16039911 | -0.00097613 | -0.61 |
| | 0.3 | -228.98561044 | -228.98432572 | 0.00239548 | -228.95667545 | -0.00111076 | -0.70 |
| | 0.4 | -228.72984241 | -228.72813568 | 0.00388880 | -228.70273151 | -0.00218207 | -1.37 |
| MP2 | 0.0 | -228.81239545 | -228.80873160 | 0.00425497 | -228.78769558 | -0.00059112 | -0.37 |
| | 0.1 | -228.76859428 | -228.76515264 | 0.00388302 | -228.74228352 | -0.00044138 | -0.28 |
| | 0.2 | -228.63905604 | -228.63583498 | 0.00369914 | -228.60909315 | -0.00047808 | -0.30 |
| | 0.3 | -228.43099072 | -228.42730691 | 0.00418627 | -228.39820774 | -0.00050246 | -0.32 |
| | 0.4 | -228.16084343 | -228.15644314 | 0.00547867 | -228.12942545 | -0.00107838 | -0.68 |
| MP3 | 0.0 | -228.82997802 | -228.82651138 | 0.00403971 | -228.80643247 | -0.00057307 | -0.36 |
| | 0.1 | -228.78672646 | -228.78347188 | 0.00368297 | -228.76166112 | -0.00042839 | -0.27 |
| | 0.2 | -228.65880461 | -228.65573612 | 0.00352865 | -228.63028608 | -0.00046016 | -0.29 |
| | 0.3 | -228.45332840 | -228.44978393 | 0.00403432 | -228.42223741 | -0.00048985 | -0.31 |
| | 0.4 | -228.18683884 | -228.18253883 | 0.00530095 | -228.15741718 | -0.00100094 | -0.63 |
| CCSD | 0.0 | -228.83422884 | -228.83073282 | 0.00408195 | -228.81045820 | -0.00058593 | -0.37 |
| | 0.1 | -228.79094323 | -228.78764975 | 0.00373234 | -228.76563378 | -0.00043886 | -0.28 |
| | 0.2 | -228.66294939 | -228.65983158 | 0.00358544 | -228.63415986 | -0.00046763 | -0.29 |
| | 0.3 | -228.45749330 | -228.45388721 | 0.00409921 | -228.42614075 | -0.00049312 | -0.31 |
| | 0.4 | -228.19144018 | -228.18706748 | 0.00538787 | -228.16181434 | -0.00101517 | -0.64 |

[a)] Electronic mail: benjamin.speake@nottingham.ac.uk

## II. WATER CLUSTER CONFORMER ERROR ANALYSIS

In these tables, the conformers of the water clusters are labelled using the notation of Ref. 1 from which the geometries are also taken.

### A. $(H_2O)_3$

TABLE S2: Energies of different conformers of $(H_2O)_3$ calculated conventionally ($E^{\mathrm{conv}}$), using EFM ($E^{\mathrm{EFM}}$) and the percentage error (including the BSSE correction) for several electronic structure methods and basis sets at zero field.

| | Method | $C_1$ | $C_{3h}$ | $C_3$ | Average |
|---|---|---|---|---|---|
| $E^{\mathrm{EFM}}$ / $E_h$ | HF/DZ | -228.13864836 | -228.13703697 | -228.13654375 | |
| | HF/TZ | -228.19626188 | -228.19493710 | -228.19420152 | |
| | MP2/DZ | -228.81172824 | -228.80837450 | -228.80900113 | |
| | MP2/TZ | -229.05618050 | -229.05283251 | -229.05343734 | |
| | TPSS/DZ | -229.36140722 | -229.35565376 | -229.35804302 | |
| | TPSS/TZ | -229.42291033 | -229.41728484 | -229.41959143 | |
| $E^{\mathrm{conv}}$ / $E_h$ | HF/DZ | -228.14014507 | -228.13989590 | -228.13926741 | |
| | HF/TZ | -228.19672790 | -228.19652019 | -228.19591629 | |
| | MP2/DZ | -228.81632439 | -228.81411677 | -228.81515169 | |
| | MP2/TZ | -229.06002449 | -229.05740762 | -229.05872980 | |
| | TPSS/DZ | -229.36234888 | -229.35860655 | -229.36073154 | |
| | TPSS/TZ | -229.42287335 | -229.41875055 | -229.42125313 | |
| Error (%) | HF/DZ | 0.00065605 | 0.00125315 | 0.00119386 | 0.00103435 |
| | HF/TZ | 0.00020422 | 0.00069374 | 0.00075145 | 0.00054980 |
| | MP2/DZ | 0.00200866 | 0.00250958 | 0.00268800 | 0.00240208 |
| | MP2/TZ | 0.00167816 | 0.00199737 | 0.00231053 | 0.00199535 |
| | TPSS/DZ | 0.00041056 | 0.00128741 | 0.00117218 | 0.00095671 |
| | TPSS/TZ | 0.00001612 | 0.00063888 | 0.00072430 | 0.00045977 |

### B. $(H_2O)_4$

TABLE S3: Energies of different conformers of $(H_2O)_4$ calculated conventionally ($E^{\text{conv}}$), using EFM ($E^{\text{EFM}}$) and the percentage error (including the BSSE correction) for several electronic structure methods and basis sets at zero field.

| | Method | $C_{4h}$ | $C_4$ | $C_i$ | $S_4$ | Average |
|---|---|---|---|---|---|---|
| $E^{\text{EFM}}$ / $E_h$ | HF/DZ | -304.18488257 | -304.18441280 | -304.18878633 | -304.18862834 | |
| | HF/TZ | -304.26187442 | -304.26091021 | -304.26513867 | -304.26506919 | |
| | MP2/DZ | -305.08093329 | -305.08235678 | -305.08827870 | -305.08795130 | |
| | MP2/TZ | -305.40708937 | -305.40830879 | -305.41414143 | -305.41400320 | |
| | TPSS/DZ | -305.81366576 | -305.81731671 | -305.82435147 | -305.82414380 | |
| | TPSS/TZ | -305.89541210 | -305.89860971 | -305.90550198 | -305.90539702 | |
| $E^{\text{conv}}$ / $E_h$ | HF/DZ | -304.19257993 | -304.19153862 | -304.19278441 | -304.19401416 | |
| | HF/TZ | -304.26845311 | -304.26724746 | -304.26835172 | -304.26954967 | |
| | MP2/DZ | -305.09438805 | -305.09617070 | -305.09823093 | -305.09966451 | |
| | MP2/TZ | -305.41987876 | -305.42169128 | -305.42373936 | -305.42518337 | |
| | TPSS/DZ | -305.82165570 | -305.82461182 | -305.82715384 | -305.82866388 | |
| | TPSS/TZ | -305.90193382 | -305.90508844 | -305.90756161 | -305.90902760 | |
| Error (%) | HF/DZ | 0.00253042 | 0.00234254 | 0.00131432 | 0.00177052 | 0.00198945 |
| | HF/TZ | 0.00216213 | 0.00208279 | 0.00105599 | 0.00147254 | 0.00169336 |
| | MP2/DZ | 0.00441003 | 0.00452772 | 0.00326198 | 0.00383914 | 0.00400972 |
| | MP2/TZ | 0.00418748 | 0.00438164 | 0.00314250 | 0.00366053 | 0.00384304 |
| | TPSS/DZ | 0.00261262 | 0.00238539 | 0.00091632 | 0.00147798 | 0.00184808 |
| | TPSS/TZ | 0.00213196 | 0.00211789 | 0.00067329 | 0.00118682 | 0.00152749 |

### C. $(H_2O)_5$

TABLE S4: Energies of different conformers of $(H_2O)_5$ calculated conventionally ($E^{\mathrm{conv}}$), using EFM ($E^{\mathrm{EFM}}$) and the percentage error (including the BSSE correction) for several electronic structure methods and basis sets at zero field.

| | Method | $C_1$ | $C_{5h}$ | $C_5$ | Average |
|---|---|---|---|---|---|
| $E^{\mathrm{EFM}}$ / $E_h$ | HF/DZ | -380.23263625 | -380.22695533 | -380.22619028 | |
| | HF/TZ | -380.32565915 | -380.32152208 | -380.31935695 | |
| | MP2/DZ | -381.35648643 | -381.34671113 | -381.34772933 | |
| | MP2/TZ | -381.76143801 | -381.75281734 | -381.75256459 | |
| | TPSS/DZ | -382.27772356 | -382.26445377 | -382.26738292 | |
| | TPSS/TZ | -382.37646780 | -382.36484196 | -382.36618335 | |
| $E^{\mathrm{conv}}$ / $E_h$ | HF/DZ | -380.24480629 | -380.24324678 | -380.24197108 | |
| | HF/TZ | -380.33940014 | -380.33828580 | -380.33682558 | |
| | MP2/DZ | -381.37759322 | -381.37141065 | -381.37290406 | |
| | MP2/TZ | -381.78446939 | -381.77882523 | -381.78001539 | |
| | TPSS/DZ | -382.28945278 | -382.28181853 | -382.28427567 | |
| | TPSS/TZ | -382.38978850 | -382.38222697 | -382.38479282 | |
| Error (%) | HF/DZ | 0.00320058 | 0.00428448 | 0.00415020 | 0.00387842 |
| | HF/TZ | 0.00361282 | 0.00440758 | 0.00459294 | 0.00420445 |
| | MP2/DZ | 0.00553435 | 0.00647650 | 0.00660108 | 0.00620398 |
| | MP2/TZ | 0.00603256 | 0.00681229 | 0.00719021 | 0.00667836 |
| | TPSS/DZ | 0.00306815 | 0.00454240 | 0.00441890 | 0.00400981 |
| | TPSS/TZ | 0.00348354 | 0.00454650 | 0.00486669 | 0.00429891 |

## D. $(H_2O)_6$

TABLE S5: Energies of different conformers of $(H_2O)_6$ calculated conventionally ($E^{\mathrm{conv}}$), using EFM ($E^{\mathrm{EFM}}$) and the percentage error (including the BSSE correction) for several electronic structure methods and basis sets at zero field.

| | Method | Bag | Boat 1 | Boat 2 | Book 1 | Book 2 | Cage | Cyclic | Prism | Average |
|---|---|---|---|---|---|---|---|---|---|---|
| $E^{\mathrm{EFM}}$ / $E_h$ | HF/DZ | -456.28334482 | -456.27792739 | -456.27567577 | -456.28481035 | -456.28801904 | -456.28643623 | -456.27336813 | -456.28432387 | |
| | HF/TZ | -456.39557930 | -456.38776402 | -456.38557514 | -456.39836920 | -456.40199370 | -456.40131347 | -456.38075161 | -456.39786437 | |
| | MP2/DZ | -457.63560410 | -457.62611486 | -457.62339695 | -457.63635113 | -457.68174798 | -457.64010253 | -457.62089943 | -457.63787268 | |
| | MP2/TZ | -458.12283681 | -458.11010728 | -458.10747348 | -458.12502664 | -458.12956832 | -458.13093727 | -458.10235162 | -458.12730837 | |
| | TPSS/DZ | -458.74019993 | -458.73151203 | -458.72857713 | -458.74114053 | -458.74566042 | -458.74332647 | -458.72608053 | -458.74041158 | |
| | TPSS/TZ | -458.85769313 | -458.84779542 | -458.84486406 | -458.86157693 | -458.86651665 | -458.86549178 | -458.83795741 | -458.86063539 | |
| $E^{\mathrm{conv}}$ / $E_h$ | HF/DZ | -456.29305242 | -456.29452629 | -456.29460447 | -456.29491873 | -456.29427573 | -456.29365180 | -456.29605192 | -456.29400197 | |
| | HF/TZ | -456.40558346 | -456.40809076 | -456.40816161 | -456.40765065 | -456.40692855 | -456.40568808 | -456.40960258 | -456.40547759 | |
| | MP2/DZ | -457.65615908 | -457.65359995 | -457.65340573 | -457.65733198 | -457.65694345 | -457.64241636 | -457.65517697 | -457.64282487 | |
| | MP2/TZ | -458.14447833 | -458.14172557 | -458.14157253 | -458.14571213 | -458.14534181 | -458.14671742 | -458.14326192 | -458.14658892 | |
| | TPSS/DZ | -458.74776537 | -458.74774076 | -458.74751161 | -458.74930082 | -458.74888067 | -458.74729347 | -458.74950355 | -458.74677408 | |
| | TPSS/TZ | -458.86746273 | -458.86803624 | -458.86778848 | -458.86923794 | -458.86881254 | -458.86703508 | -458.86975889 | -458.86621862 | |
| Error (%) | HF/DZ | 0.00212749 | 0.00363776 | 0.00414835 | 0.00221532 | 0.00137120 | 0.00158134 | 0.00497129 | 0.00212102 | 0.00277172 |
| | HF/TZ | 0.00219195 | 0.00445363 | 0.00494874 | 0.00203359 | 0.00108124 | 0.00095849 | 0.00632129 | 0.00166808 | 0.00295713 |
| | MP2/DZ | 0.00449136 | 0.00600565 | 0.00655710 | 0.00458440 | 0.00541990 | 0.00050560 | 0.00748982 | 0.00108211 | 0.00451699 |
| | MP2/TZ | 0.00472373 | 0.00690142 | 0.00744291 | 0.00451505 | 0.00344290 | 0.00344434 | 0.00892959 | 0.00420838 | 0.00545104 |
| | TPSS/DZ | 0.00164915 | 0.00353761 | 0.00412743 | 0.00177881 | 0.00070196 | 0.00086475 | 0.00510584 | 0.00138693 | 0.00239406 |
| | TPSS/TZ | 0.00212907 | 0.00441103 | 0.00499587 | 0.00166954 | 0.00050034 | 0.00033633 | 0.00693039 | 0.00121674 | 0.00277366 |

## E. $(H_2O)_7$

TABLE S6: Energies of different conformers of $(H_2O)_7$ calculated conventionally ($E^{\rm conv}$), using EFM ($E^{\rm EFM}$) and the percentage error (including the BSSE correction) for several electronic structure methods and basis sets at zero field.

| | Method | A | B | CA1 | CA2 | CH1 | CH2 | CH3 | C | D | PR2 | PR3 | Average |
|---|---|---|---|---|---|---|---|---|---|---|---|---|---|
| $E^{\rm EFM}$ / $E_h$ | HF/DZ | -532.33502047 | -532.33479426 | -532.33403725 | -532.33277298 | -532.32784619 | -532.32931832 | -532.32783583 | -532.32759812 | -532.33389252 | -532.33388491 | -532.33188583 | |
| | HF/TZ | -532.46741003 | -532.46798034 | -532.46656915 | -532.46471215 | -532.45700202 | -532.45805402 | -532.45698733 | -532.45636939 | -532.46797767 | -532.46638277 | -532.46395873 | |
| | MP2/DZ | -533.91542701 | -533.91403631 | -533.91312817 | -533.91187361 | -533.90371147 | -533.90565964 | -533.90370974 | -533.90349881 | -533.91136847 | -533.91415823 | -533.91108682 | |
| | MP2/TZ | -534.48655018 | -534.48570420 | -534.48397062 | -534.48207494 | -534.47024668 | -534.47171343 | -534.47023851 | -534.46960345 | -534.48316322 | -534.48545657 | -534.48182112 | |
| | TPSS/DZ | -535.20313689 | -535.20200854 | -535.20169362 | -535.19910988 | -535.19318778 | -535.19538618 | -535.19318866 | -535.19287741 | -535.20112169 | -535.20257925 | -535.19725977 | |
| | TPSS/TZ | -535.34261968 | -535.34307054 | -535.34132251 | -535.33788240 | -535.32737567 | -535.32881438 | -535.32736238 | -535.32621794 | -535.34394586 | -535.34285752 | -535.33554094 | |
| $E^{\rm conv}$ / $E_h$ | HF/DZ | -532.34711961 | -532.34549025 | -532.34599439 | -532.34448606 | -532.34555702 | -532.34603544 | -532.34555072 | -532.34538936 | -532.34436231 | -532.34649797 | -532.34676418 | |
| | HF/TZ | -532.47741227 | -532.47649496 | -532.47683588 | -532.47537157 | -532.47731096 | -532.47775274 | -532.47730335 | -532.47716313 | -532.47599865 | -532.47671739 | -532.47717182 | |
| | MP2/DZ | -533.94161766 | -533.93841357 | -533.93877478 | -533.93738645 | -533.93511734 | -533.93575967 | -533.93511875 | -533.93494324 | -533.93476588 | -533.94089849 | -533.94057647 | |
| | MP2/TZ | -534.51146059 | -534.50863242 | -534.50882702 | -534.50757201 | -534.50499220 | -534.50559245 | -534.50499045 | -534.50480206 | -534.50485188 | -534.51094882 | -534.51044754 | |
| | TPSS/DZ | -535.21181413 | -535.21022165 | -535.21059898 | -535.20917665 | -535.20993926 | -535.21056371 | -535.20994378 | -535.20968190 | -535.20906270 | -535.21134405 | -535.21062790 | |
| | TPSS/TZ | -535.35097701 | -535.34981827 | -535.34990076 | -535.34858614 | -535.34984912 | -535.35042750 | -535.34984973 | -535.34959767 | -535.34917609 | -535.35049160 | -535.34983875 | |
| Error (%) | HF/DZ | 0.00227279 | 0.00200922 | 0.00224612 | 0.00220028 | 0.00332694 | 0.00314027 | 0.00332770 | 0.00334205 | 0.00196673 | 0.00236933 | 0.00279486 | 0.00263603 |
| | HF/TZ | 0.00187843 | 0.00159906 | 0.00192811 | 0.00200186 | 0.00381405 | 0.00369944 | 0.00381538 | 0.00390510 | 0.00150636 | 0.00194086 | 0.00248144 | 0.00259728 |
| | MP2/DZ | 0.00490515 | 0.00456556 | 0.00480329 | 0.00477825 | 0.00588196 | 0.00563739 | 0.00588255 | 0.00588919 | 0.00438207 | 0.00500809 | 0.00552302 | 0.00520514 |
| | MP2/TZ | 0.00466041 | 0.00428959 | 0.00465033 | 0.00477020 | 0.00650050 | 0.00633838 | 0.00650170 | 0.00658528 | 0.00405771 | 0.00476927 | 0.00535563 | 0.00531627 |
| | TPSS/DZ | 0.00162127 | 0.00153456 | 0.00166390 | 0.00188090 | 0.00312989 | 0.00283581 | 0.00313057 | 0.00313980 | 0.00148372 | 0.00163763 | 0.00249773 | 0.00223234 |
| | TPSS/TZ | 0.00156109 | 0.00126043 | 0.00160236 | 0.00199940 | 0.00419790 | 0.00403719 | 0.00420050 | 0.00436719 | 0.00097698 | 0.00142600 | 0.00267074 | 0.00257271 |

### F. $(H_2O)_8$

TABLE S7: Energies of different conformers of $(H_2O)_8$ calculated conventionally ($E^{\text{conv}}$), using EFM ($E^{\text{EFM}}$) and the percentage error (including the BSSE correction) for several electronic structure methods and basis sets at zero field.

| | Method | $C_1$a | $C_1$b | $C_1$c | $C_2$ | $C_i$ | $C_s$ | $D_{2d}$ | Noncubic 1 | $S_4$ | Average |
|---|---|---|---|---|---|---|---|---|---|---|---|
| $E^{\text{EFM}}$ / $E_h$ | HF/DZ | -608.38412515 | -608.38564910 | -608.38880259 | -608.38669874 | -608.39131481 | -608.38826991 | -608.38287612 | -608.38416176 | -608.38786421 | |
| | HF/TZ | -608.53584031 | -608.53778935 | -608.54207329 | -608.53858432 | -608.54470785 | -608.54034531 | -608.53369623 | -608.53593855 | -608.54112486 | |
| | MP2/DZ | -610.19304383 | -610.19484429 | -610.19794107 | -610.19683664 | -610.20170739 | -610.19838979 | -610.19223357 | -610.19173747 | -610.19747825 | |
| | MP2/TZ | -610.84700839 | -610.84931629 | -610.85346551 | -610.85121880 | -610.85762895 | -610.85282554 | -610.84552708 | -610.84553675 | -610.85340324 | |
| | TPSS/DZ | -611.66606766 | -611.66748921 | -611.67134176 | -611.67109697 | -611.67590667 | -611.67091425 | -611.66506249 | -611.66481610 | -611.67143014 | |
| | TPSS/TZ | -611.82612957 | -611.82668709 | -611.83378226 | -611.83210731 | -611.83880063 | -611.82661713 | -611.82271618 | -611.82479732 | -611.83366548 | |
| $E^{\text{conv}}$ / $E_h$ | HF/DZ | -608.39891470 | -608.39873856 | -608.39884218 | -608.39977219 | -608.39963946 | -608.39821056 | -608.40334563 | -608.39871315 | -608.40325498 | |
| | HF/TZ | -608.54732978 | -608.54715859 | -608.54724178 | -608.54814806 | -608.54801259 | -608.54659704 | -608.55166985 | -608.54760890 | -608.55157010 | |
| | MP2/DZ | -610.22490886 | -610.22507122 | -610.20361134 | -610.22685108 | -610.20550952 | -610.20408559 | -610.23165148 | -610.22336527 | -610.21034296 | |
| | MP2/TZ | -610.87684521 | -610.87701131 | -610.87671503 | -610.87882194 | -610.87871028 | -610.87730285 | -610.88367050 | -610.87523421 | -610.88356412 | |
| | TPSS/DZ | -611.67568930 | -611.67596785 | -611.67565276 | -611.67781738 | -611.67776349 | -611.67634238 | -611.68219457 | -611.67477892 | -611.66473085 | |
| | TPSS/TZ | -611.83439981 | -611.83464413 | -611.83432232 | -611.83647176 | -611.83638583 | -611.83496762 | -611.84078648 | -611.83364870 | -611.84072429 | |
| Error (%) | HF/DZ | 0.00243090 | 0.00215146 | 0.00165017 | 0.00214883 | 0.00136829 | 0.00163391 | 0.00336446 | 0.00239175 | 0.00252970 | 0.00218550 |
| | HF/TZ | 0.00188802 | 0.00153961 | 0.00084932 | 0.00157157 | 0.00054305 | 0.00102732 | 0.00295351 | 0.00191774 | 0.00171641 | 0.00155628 |
| | MP2/DZ | 0.00522185 | 0.00495341 | 0.00092924 | 0.00491857 | 0.00062309 | 0.00093343 | 0.00645950 | 0.00518299 | 0.00210824 | 0.00348115 |
| | MP2/TZ | 0.00488426 | 0.00453365 | 0.00380593 | 0.00451860 | 0.00345099 | 0.00400691 | 0.00624397 | 0.00486146 | 0.00493725 | 0.00458256 |
| | TPSS/DZ | 0.00157300 | 0.00138613 | 0.00070479 | 0.00109868 | 0.00030356 | 0.00088742 | 0.00280081 | 0.00162878 | 0.00109526 | 0.00127538 |
| | TPSS/TZ | 0.00135171 | 0.00130052 | 0.00008827 | 0.00071334 | 0.00039468 | 0.00136483 | 0.00295343 | 0.00144670 | 0.00115370 | 0.00119635 |

### G. $(H_2O)_9$

TABLE S8: Energies of different conformers of $(H_2O)_9$ calculated conventionally ($E^{\mathrm{conv}}$), using EFM ($E^{\mathrm{EFM}}$) and the percentage error (including the BSSE correction) for several electronic structure methods and basis sets at zero field.

| | Method | $D_{2d}$Dah | $D_{2d}$DDh | $S_4$Dah 1 | $S_4$Dah 2 | $S_4$Danh 1 | $S_4$Danh 2 | $S_4$DDh 1 | $S_4$DDh 2 | Average |
|---|---|---|---|---|---|---|---|---|---|---|
| $E^{\mathrm{EFM}}$ / $E_h$ | HF/DZ | -684.43081661 | -684.42572765 | -684.43604828 | -684.43617710 | -684.43677913 | -684.43445021 | -684.43449784 | -684.43421446 | |
| | HF/TZ | -684.59900896 | -684.59016733 | -684.60679770 | -684.60728937 | -684.60823431 | -684.60572179 | -684.60490842 | -684.60454673 | |
| | MP2/DZ | -686.46618318 | -686.45981888 | -686.47140733 | -686.47153434 | -686.47275447 | -686.46968240 | -686.46951484 | -686.46902516 | |
| | MP2/TZ | -687.19922930 | -687.18873632 | -687.20717383 | -687.20776693 | -687.20925435 | -687.20599813 | -687.20490650 | -687.20429178 | |
| | TPSS/DZ | -688.12287315 | -688.11607889 | -688.12989242 | -688.12998091 | -688.13201151 | -688.12850058 | -688.12863708 | -688.12796509 | |
| | TPSS/TZ | -688.29654658 | -688.27819553 | -688.30991157 | -688.31063335 | -688.31357473 | -688.30974254 | -688.30820752 | -688.30731500 | |
| $E^{\mathrm{conv}}$ / $E_h$ | HF/DZ | -684.45338531 | -684.45477776 | -684.45384416 | -684.45390424 | -684.45334426 | -684.45329758 | -684.45417356 | -684.45415926 | |
| | HF/TZ | -684.62069685 | -684.62210701 | -684.62111841 | -684.62119008 | -684.62064370 | -684.62061360 | -684.62149020 | -684.62148363 | |
| | MP2/DZ | -686.51025409 | -686.51117296 | -686.51075396 | -686.51074245 | -686.51029829 | -686.50999337 | -686.51054697 | -686.51053806 | |
| | MP2/TZ | -687.24366831 | -687.24447295 | -687.24402960 | -687.24400519 | -687.24365866 | -687.24335053 | -687.24383935 | -687.24387250 | |
| | TPSS/DZ | -688.14339688 | -688.14422202 | -688.14388748 | -688.14383100 | -688.14344682 | -688.14315757 | -688.14369080 | -688.14369726 | |
| | TPSS/TZ | -688.32189876 | -688.32254816 | -688.32222985 | -688.32217701 | -688.32190552 | -688.32162752 | -688.32207011 | -688.32209212 | |
| Error (%) | HF/DZ | 0.00329733 | 0.00424427 | 0.00260001 | 0.00258997 | 0.00242020 | 0.00275364 | 0.00287466 | 0.00291397 | 0.00296176 |
| | HF/TZ | 0.00316787 | 0.00466530 | 0.00209177 | 0.00203042 | 0.00181259 | 0.00217519 | 0.00242204 | 0.00247391 | 0.00260489 |
| | MP2/DZ | 0.00641956 | 0.00748044 | 0.00573139 | 0.00571122 | 0.00546879 | 0.00587187 | 0.00597691 | 0.00604694 | 0.00608839 |
| | MP2/TZ | 0.00646627 | 0.00811016 | 0.00536284 | 0.00527298 | 0.00500613 | 0.00543510 | 0.00566507 | 0.00575934 | 0.00588474 |
| | TPSS/DZ | 0.00298248 | 0.00408971 | 0.00203374 | 0.00201267 | 0.00166176 | 0.00212993 | 0.00218758 | 0.00228617 | 0.00242301 |
| | TPSS/TZ | 0.00368319 | 0.00644358 | 0.00178961 | 0.00167707 | 0.00121030 | 0.00172666 | 0.00201397 | 0.00214683 | 0.00258640 |

## H. $(H_2O)_{10}$

TABLE S9: Energies of different conformers of $(H_2O)_{10}$ calculated conventionally ($E^{conv}$), using EFM ($E^{EFM}$) and the percentage error (including the BSSE correction) for several electronic structure methods and basis sets at zero field.

| | Method | DP1 | DP2 | DP3 | DP4 | DP5 | DP6 | DP7 | DP8 | OB1 | OB2 | OB3 | |
|---|---|---|---|---|---|---|---|---|---|---|---|---|---|
| $E^{EFM}$ / $E_h$ | HF/DZ | -760.47376139 | -760.48107410 | -760.48471716 | -760.47342924 | -760.48151971 | -760.48727935 | -760.48002547 | -760.48103670 | -760.47461476 | -760.48153637 | -760.48193788 | |
| | HF/TZ | -760.65515494 | -760.66715492 | -760.67290374 | -760.65503661 | -760.66939870 | -760.67668820 | -760.66741797 | -760.66886106 | -760.66023587 | -760.67138715 | -760.67177558 | |
| | MP2/DZ | -762.73413523 | -762.74197618 | -762.74627306 | -762.73359687 | -762.74204089 | -762.74969007 | -762.73946177 | -762.74128420 | -762.73553208 | -762.74318555 | -762.74361676 | |
| | MP2/TZ | -763.54242246 | -763.55541063 | -763.56190454 | -763.54218329 | -763.55727365 | -763.56660405 | -763.55408004 | -763.55635273 | -763.54878533 | -763.56114724 | -763.56155253 | |
| | TPSS/DZ | -764.56968278 | -764.58213107 | -764.58993742 | -764.57384141 | -764.58626558 | -764.59424265 | -764.58260081 | -764.58530332 | -764.57339035 | -764.58563187 | -764.58605859 | |
| | TPSS/TZ | -764.74604350 | -764.77407342 | -764.78789942 | -764.75336556 | -764.78369802 | -764.79455832 | -764.77913725 | -764.78244072 | -764.76485277 | -764.78579560 | -764.78618691 | |
| $E^{conv}$ / $E_h$ | HF/DZ | -760.50531878 | -760.50532797 | -760.50452233 | -760.50464516 | -760.50505202 | -760.50413427 | -760.50516036 | -760.50478815 | -760.50549111 | -760.50523625 | -760.50526662 | |
| | HF/TZ | -760.69120160 | -760.69120183 | -760.69065902 | -760.45292707 | -760.69130847 | -760.69030139 | -760.69152622 | -760.69108428 | -760.69086991 | -760.69062961 | -760.69064548 | |
| | MP2/DZ | -762.79071749 | -762.79074944 | -762.78992175 | -762.59683850 | -762.78950459 | -762.78971678 | -762.78924459 | -762.78910239 | -762.79167336 | -762.79151970 | -762.79152605 | |
| | MP2/TZ | -763.60552397 | -763.60554417 | -763.60480774 | -763.41057390 | -763.60451075 | -763.60467589 | -763.60422973 | -763.60414333 | -763.60646580 | -763.60633664 | -763.60630718 | |
| | TPSS/DZ | -764.60506424 | -764.60505918 | -764.60581759 | -764.60530038 | -764.60544800 | -764.60561482 | -764.60532108 | -764.60506107 | -764.60421766 | -764.60417256 | -764.60416494 | |
| | TPSS/TZ | -764.80324859 | -764.80318471 | -764.80392641 | -764.62879820 | -764.80366033 | -764.80381324 | -764.80359331 | -764.80331419 | -764.80226812 | -764.80222255 | -764.80219316 | |
| Error (%) | HF/DZ | 0.00414953 | 0.00318918 | 0.00260422 | 0.00410463 | 0.00309430 | 0.00221628 | 0.00330503 | 0.00312312 | 0.00405998 | 0.00311633 | 0.00306753 | |
| | HF/TZ | 0.00473867 | 0.00316119 | 0.00233410 | 0.02657752 | 0.00288024 | 0.00178958 | 0.00316925 | 0.00292145 | 0.00402713 | 0.00252960 | 0.00248063 | |
| | MP2/DZ | 0.00741780 | 0.00639406 | 0.00572224 | 0.01793325 | 0.00622239 | 0.00524741 | 0.00652642 | 0.00626886 | 0.00735998 | 0.00633648 | 0.00628078 | |
| | MP2/TZ | 0.00826363 | 0.00656537 | 0.00561851 | 0.01723966 | 0.00618607 | 0.00498581 | 0.00656750 | 0.00625856 | 0.00755369 | 0.00591789 | 0.00586096 | |
| | TPSS/DZ | 0.00462742 | 0.00299869 | 0.00207691 | 0.00411441 | 0.00250880 | 0.00148733 | 0.00297150 | 0.00258405 | 0.00403180 | 0.00242487 | 0.00236807 | |
| | TPSS/TZ | 0.00747971 | 0.00380638 | 0.00209557 | 0.01629122 | 0.00261012 | 0.00121010 | 0.00319769 | 0.00272926 | 0.00489216 | 0.00214787 | 0.00209286 | |
| | Method | OB4 | OB5 | OB6 | OB7 | OB8 | OB9 | PP1 | PP2 | PP3 | PP4 | PP5 | Average |
| $E^{EFM}$ / $E_h$ | HF/DZ | -760.47722245 | -760.47462497 | -760.47914374 | -760.48862006 | -760.48433713 | -760.49022187 | -760.47023807 | -760.48069519 | -760.48299400 | -760.49117065 | -760.48456933 | |
| | HF/TZ | -760.66598733 | -760.66025301 | -760.66787230 | -760.68096478 | -760.67496228 | -760.68112967 | -760.64885999 | -760.66794308 | -760.67134646 | -760.68242530 | -760.67338044 | |
| | MP2/DZ | -762.73873081 | -762.73553853 | -762.74112336 | -762.75200425 | -762.74742565 | -762.75353815 | -762.73121821 | -762.74203450 | -762.74613991 | -762.75513628 | -762.74764190 | |
| | MP2/TZ | -763.55556145 | -763.54879951 | -763.55793705 | -763.57292929 | -763.56648370 | -763.57269498 | -763.53700828 | -763.55723460 | -763.56264457 | -763.57475372 | -763.56456560 | |
| | TPSS/DZ | -764.57800404 | -764.57339431 | -764.58128774 | -764.59589683 | -764.59139654 | -764.59647429 | -764.56734273 | -764.58485365 | -764.58972415 | -764.59903789 | -764.59136762 | |
| | TPSS/TZ | -764.77338371 | -764.76488511 | -764.77691832 | -764.80091058 | -764.79444492 | -764.79802428 | -764.73556415 | -764.78079664 | -764.78746544 | -764.80143693 | -764.79053348 | |
| $E^{conv}$ / $E_h$ | HF/DZ | -760.50420878 | -760.50549736 | -760.50419832 | -760.50429717 | -760.50430266 | -760.50401150 | -760.50708825 | -760.50703135 | -760.50584459 | -760.50567071 | -760.50592757 | |
| | HF/TZ | -760.68972538 | -760.69087728 | -760.68964950 | -760.68978673 | -760.68982928 | -760.68937453 | -760.69283349 | -760.69272670 | -760.69156238 | -760.69136620 | -760.69160795 | |
| | MP2/DZ | -762.79073382 | -762.79167235 | -762.79081191 | -762.79031984 | -762.79030924 | -762.79005717 | -762.79411839 | -762.79403994 | -762.79310945 | -762.79302300 | -762.79300047 | |
| | MP2/TZ | -763.60567089 | -763.60646555 | -763.60567177 | -763.60522657 | -763.60528246 | -763.60496412 | -763.60933766 | -763.60915156 | -763.60841898 | -763.60830229 | -763.60817707 | |
| | TPSS/DZ | -764.60345517 | -764.60421555 | -764.60340226 | -764.60328445 | -764.60322184 | -764.60302968 | -764.60817841 | -764.60820783 | -764.60725055 | -764.60724370 | -764.60713979 | |
| | TPSS/TZ | -764.80175190 | -764.80226656 | -764.80162402 | -764.80148578 | -764.80147458 | -764.80105692 | -764.80614663 | -764.80611734 | -764.80539488 | -764.80535360 | -764.80523673 | |
| Error (%) | HF/DZ | 0.00354848 | 0.00405946 | 0.00329447 | 0.00206141 | 0.00262530 | 0.00181322 | 0.00484547 | 0.00346297 | 0.00300466 | 0.00190663 | 0.00280843 | 0.00315730 |
| | HF/TZ | 0.00312060 | 0.00402585 | 0.00286282 | 0.00115973 | 0.00195441 | 0.00108387 | 0.00578072 | 0.00325803 | 0.00265757 | 0.00117536 | 0.00239618 | 0.00391293 |
| | MP2/DZ | 0.00681747 | 0.00735900 | 0.00651405 | 0.00502308 | 0.00562194 | 0.00478756 | 0.00824602 | 0.00681776 | 0.00615757 | 0.00496684 | 0.00594638 | 0.00681670 |
| | MP2/TZ | 0.00656221 | 0.00755180 | 0.00625123 | 0.00422958 | 0.00508100 | 0.00422589 | 0.00947204 | 0.00679889 | 0.00599449 | 0.00439343 | 0.00571124 | 0.00669497 |
| | TPSS/DZ | 0.00332867 | 0.00403101 | 0.00289229 | 0.00096620 | 0.00154659 | 0.00085736 | 0.00534073 | 0.00305440 | 0.00229221 | 0.00107321 | 0.00206278 | 0.00271088 |
| | TPSS/TZ | 0.00370922 | 0.00488773 | 0.00323034 | 0.00007521 | 0.00091915 | 0.00039653 | 0.00922881 | 0.00331073 | 0.00234431 | 0.00051211 | 0.00192248 | 0.00359498 |

## III. GEOMETRY OF $(H_2O)_{103}$

```
309

O     -9.655737     -2.846434      1.540634
H    -10.059320     -3.161828      2.349018
H     -9.131738     -2.093068      1.812124
O      4.099744      4.505032      1.673771
H      3.570766      4.879393      2.377953
H      3.585243      4.655800      0.881054
O     -0.440099      0.230832      0.983328
H     -0.579147     -0.716006      0.982968
H     -0.995654      0.554657      1.692086
O     -0.866651      4.692060     -2.397008
H     -0.413843      3.850929     -2.454452
H     -0.164704      5.337309     -2.314747
O     -2.722841      0.191639     -7.116122
H     -3.321996     -0.420366     -7.543087
H     -2.507929      0.830423     -7.795542
O      0.509574      6.991268     -1.965288
H      0.023507      7.775772     -2.218526
H      1.056229      6.792011     -2.725095
O     -8.263569     -2.849367     -5.206974
H     -8.694101     -2.222274     -4.626255
H     -8.361999     -3.692438     -4.764952
O     -0.246280     -2.863948      1.848094
H      0.593108     -2.492494      2.118810
H     -0.085145     -3.805636      1.792401
O     -8.043692     -1.168501      3.209565
H     -7.148035     -1.290777      2.895425
H     -8.044219     -1.549682      4.087371
O     -8.522794      3.549999      4.708282
H     -8.160924      3.946705      5.500438
H     -7.756053      3.276941      4.204904
O     -5.381594     -0.741515     -3.683368
H     -5.753232     -0.393548     -2.873033
H     -5.161361      0.034155     -4.198805
O     -4.455829      7.583134     -6.560235
H     -4.598189      7.488289     -7.501816
H     -4.267060      8.513413     -6.438619
O    -11.360351     -2.536454     -0.598540
H    -12.185848     -2.534847     -0.114402
H    -10.695097     -2.728265      0.062132
O     -3.893035      7.564836      6.265499
H     -4.175474      6.700026      6.562432
H     -3.026188      7.417531      5.887717
O     -8.775303     -3.856553     -0.887635
H     -9.105467     -3.868569      0.010522
H     -8.205145     -3.088775     -0.923424
O     -6.732769     -6.532250      2.992206
H     -6.978275     -7.453251      3.077769
H     -7.350619     -6.065962      3.554948
O     -7.467966      6.200736     -2.266136
H     -7.307404      6.053799     -1.334221
H     -8.313691      5.784860     -2.432388
O     -7.387840      8.366459     -3.818464
H     -6.517457      8.463833     -4.204195
H     -7.300092      7.632684     -3.210422
O     -4.397440      2.468101     -9.453060
H     -5.240707      2.499779     -9.001691
H     -4.559036      1.927032    -10.225700
O     -7.696522      5.395679      0.785185
H     -6.927583      4.890630      0.521566
H     -8.427332      4.784311      0.695806
O     -6.132602      5.437133     -6.223101
H     -7.021913      5.445131     -6.576512
H     -5.857914      6.353625     -6.243861
O     -4.949812     -5.023650     -5.146999
H     -4.809796     -4.870058     -4.212846
H     -4.979584     -4.148145     -5.532288
O     -5.392600     -0.242917      5.597418
H     -4.878279      0.451469      6.008683
H     -5.161350     -0.195586      4.669988
O     -6.310768      2.829478      3.398714
H     -6.746881      2.315655      2.719282
H     -5.464209      2.399537      3.518424
O     -3.645205      1.790396      5.300281
H     -3.046875      1.458153      5.969201
H     -3.247740      1.526829      4.470585
O     -5.251134     -2.436139      2.686985
H     -5.468988     -3.227659      2.195179
H     -5.285722     -2.706517      3.604341
O     -1.977169     -2.071454      3.713954
H     -2.256253     -1.157023      3.671934
H     -1.426746     -2.190127      2.940136
O     -6.908806     -5.713591     -0.746942
H     -7.279856     -6.569274     -0.961335
H     -7.621461     -5.095691     -0.908695
O     -4.716718      0.452327      2.348370
H     -5.524076      0.671625      1.883700
H     -4.733952     -0.501660      2.422233
O     -6.246147      7.419488      3.772843
H     -5.705325      7.756751      3.058979
H     -5.920924      6.531810      3.921504
O      0.470771      0.728271     -2.712423
H      1.217363      0.130357     -2.743023
H     -0.127495      0.399597     -3.383164
O     -5.134405      9.837667     -1.769595
H     -4.308085      9.757022     -1.293646
H     -5.788883      9.465539     -1.178814
O     -5.787133     -4.737356      1.220867
H     -6.008038     -5.394789      1.880275
H     -6.255796     -5.018553      0.435293
O    -10.669477      5.149336      5.143401
H     -9.981157      4.484506      5.135996
H    -10.303594      5.879086      4.643964
O     -8.267619      2.047430     -1.828547
H     -8.505854      1.284951     -2.355527
H     -7.522402      2.433526     -2.288355
O     -4.620080     -1.596362     -8.222754
H     -4.992429     -1.933923     -7.408354
H     -5.197022     -1.933060     -8.908038
O     -4.581709     -0.271894     -1.073007
H     -4.165035      0.589626     -1.074972
H     -3.992522     -0.822204     -0.557392
O     -3.186622     -4.991106      0.342772
H     -2.926760     -5.684165      0.949384
H     -4.120948     -4.865821      0.507637
O     -4.343625      4.317154      6.018975
H     -4.231362      3.379478      6.173891
H     -5.269901      4.480330      6.195690
O    -11.008226      4.032661     -6.460134
H    -11.812864      4.464630     -6.174133
H    -10.572521      3.774980     -5.647973
O     -3.053414     -2.132401      0.966603
H     -2.780937     -2.992481      0.647455
H     -3.613325     -2.322069      1.719178
O    -10.049478      5.075029     -1.841895
H    -10.100136      4.791784     -0.929183
H    -10.803556      5.653136     -1.955919
O      1.321089     -3.090371     -1.453464
H      1.061003     -2.361663     -0.890278
H      0.537057     -3.635302     -1.518162
O     -8.596769      0.747015     -5.631122
H     -9.242221      1.323573     -6.039545
H     -7.789901      1.261437     -5.617862
O     -2.610418      2.894085     -3.976592
H     -1.820950      3.432337     -3.923093
H     -3.174568      3.356514     -4.596057
O     -3.367634      6.523115      1.514739
H     -3.548173      5.732449      2.022766
H     -4.214859      6.962802      1.445987
O     -6.808889     -1.936732     -0.849072
H     -5.948508     -1.542280     -0.990460
H     -7.429401     -1.273361     -1.150316
O     -1.124678      5.073390     -5.922418
H     -1.966313      4.618600     -5.896836
H     -0.978202      5.255001     -6.850537
O     -3.095105      6.207848     -2.111108
H     -2.261916      5.805664     -2.355817
H     -3.628394      6.156460     -2.904080
O     -4.787088      1.004197     -5.666391
H     -3.958970      0.894350     -6.133286
H     -5.448885      1.039691     -6.356758
O     -5.451346      5.442486     -3.705255
H     -6.243578      5.759192     -3.271777
H     -5.633465      5.540762     -4.639609
O     -7.126732      5.076740      6.780423
H     -8.013363      5.005451      7.133465
H     -6.904821      6.002902      6.874356
O      0.344882     -0.490874     -7.205944
H      0.848834     -0.400621     -6.397412
H     -0.476783     -0.898121     -6.932338
O     -5.509882      7.891874      1.058528
H     -5.894312      8.690520      0.697674
H     -6.043074      7.184273      0.696798
O     -9.711556     -0.155951     -0.305286
H    -10.460158     -0.743963     -0.403648
H     -9.417257      0.009659     -1.200721
O    -10.066205      3.404861      0.268345
H    -10.268073      2.880394      1.042955
H     -9.974884      2.765071     -0.437468
O      2.484935      5.332527     -3.018302
H      1.994008      4.752357     -3.599878
H      3.367957      4.963987     -3.001221
O     -1.518760      6.913785      5.195422
H     -0.738206      7.179043      5.681436
H     -1.445670      7.369307      4.356971
O     -5.601233     -2.509608     -5.874882
H     -5.516417     -1.927517     -5.120022
H     -6.535779     -2.710945     -5.918804
O      0.758682      2.093326     -0.442857
H      0.387260      1.630981      0.308223
H      0.489886      1.574590     -1.200817
O      2.541224     -0.961072     -2.424610
H      3.044017     -0.759596     -1.635660
H      2.092646     -1.781362     -2.220295
O     -6.808812     10.081662      0.137232
H     -6.903219     10.497205      0.994116
H     -7.677540     10.148110     -0.258670
O    -11.441106      7.719646      1.007046
H    -11.597840      8.202338      1.818391
H    -10.798728      7.051593      1.245576
O     -5.013110     -4.342279     -2.304820
H     -5.559726     -4.989410     -1.859546
H     -5.412533     -3.500302     -2.087153
O     -9.438331      3.622615     -4.080618
H     -8.567196      3.280774     -4.280881
H     -9.327929      4.091088     -3.253462
O    -10.956082      2.409737      2.938047
H    -10.483888      2.957522      3.564785
H    -11.823682      2.295298      3.325366
O     -2.720823      3.617007      0.405587
H     -2.347652      4.301517     -0.149414
H     -2.718153      2.833995     -0.144620
O      1.443997      1.513800      3.318267
H      0.751444      1.967823      2.838611
H      1.476779      1.954724      4.167004
O     -5.329539      5.134232     -0.280266
H     -4.563543      4.901880      0.244233
H     -4.968911      5.574254     -1.049793
O     -7.403043      1.068413      1.306596
H     -7.528716      1.019361      0.359159
H     -8.138364      0.576813      1.671940
O     -4.890675      5.050539      3.427871
H     -5.555144      4.392536      3.224524
H     -4.672825      4.895537      4.346761
O     -1.531059      7.871756     -5.594979
H     -2.426668      7.653226     -5.851818
H     -1.081856      7.027964     -5.549428
O      1.073268      3.055470     -3.950185
H      1.516300      2.872106     -4.778398
H      0.755862      2.202639     -3.653916
O     -3.491496     -6.994281     -1.728638
H     -3.294175     -7.442941     -0.906685
H     -3.745481     -6.110230     -1.464465
O    -10.857310     -2.192634     -3.579144
H    -11.284234     -2.867915     -4.105992
H    -11.025224     -2.452899     -2.673655
O     -2.626148      1.493572     -1.768487
H     -2.758960      2.003836     -2.567129
H     -1.826088      0.994795     -1.932703
O      2.641529      0.111520     -5.173280
H      3.151472     -0.124701     -5.947873
H      3.008315     -0.425881     -4.471489
O     -8.563846     -1.061338     -8.670604
H     -8.439551     -0.998222     -7.723818
H     -9.300566     -1.662930     -8.776304
O      3.011054      0.221263      0.150742
H      2.314587      0.870731      0.245419
H      3.821007      0.724172      0.233822
O     -8.860910      3.471269     -7.872709
H     -8.674925      4.406622     -7.952469
H     -9.804651      3.426419     -7.720446
O     -2.187563      0.978336      2.874415
H     -1.895358      1.856785      2.631968
H     -2.962089      0.825307      2.333551
O     -1.099822      7.649145      2.687171
H     -1.937038      7.328233      2.352624
H     -0.514873      7.627888      1.930056
O     -9.336178      7.219470      3.744530
H     -8.437553      7.509804      3.899501
H     -9.768187      7.976564      3.349537
O     -2.166636      0.741756      7.237086
H     -1.343926      0.307087      7.460801
H     -2.845453      0.166690      7.589719
O     -2.241657      3.787322     -8.569861
H     -2.911119      3.340768     -9.087788
H     -1.432577      3.314952     -8.765049
O      2.144533     -1.188709      2.261552
H      2.305467     -0.586078      1.535761
H      1.884495     -0.624027      2.989120
O     -0.781264      3.199612      2.296120
H     -1.315779      3.616393      1.620527
H     -0.922854      3.733694      3.077498
O     -6.977678      1.603609     -8.171402
H     -7.558371      2.305800     -7.878895
H     -7.559677      0.969730     -8.590111
O     -6.357434      2.640724     -3.982986
H     -5.679121      2.196027     -4.490897
H     -6.134773      3.569286     -4.046536
O     -8.798847     -4.726403     -3.469670
H     -8.965435     -4.436861     -2.572869
H     -8.419991     -5.599917     -3.373311
O     -9.950103      8.880328     -2.831155
H     -9.179828      8.569025     -3.306133
H     -9.596998      9.276700     -2.034883
O     -4.830113     -3.079291      5.256129
H     -4.106499     -3.492824      5.726459
H     -4.894114     -2.203454      5.636465
O     -1.785040      4.268522      4.797496
H     -2.691758      4.338296      5.095539
H     -1.466289      5.170796      4.785568
O      2.040154      4.481204     -0.326943
H      1.556631      3.657419     -0.385426
H      2.286913      4.679220     -1.230128
O     -2.085942     -0.899877     -3.727699
H     -2.433838     -0.186847     -4.262857
H     -2.624760     -1.656832     -3.956907
O     -2.550034      8.437691     -0.444647
H     -2.872679      7.876326      0.260056
H     -2.564186      7.879336     -1.221745
O     -8.741728     -0.261625     -3.151150
H     -8.564313     -0.022275     -4.060586
H     -9.459316     -0.892530     -3.204701
O     -7.601083     -1.854852      5.706927
H     -6.748722     -1.481145      5.929773
H     -7.734150     -2.549929      6.351135
O     -3.634262      4.032822     -6.167898
H     -4.394482      4.544622     -6.443513
H     -3.334898      3.598765     -6.966533
O     -1.134748     -4.351545      4.920190
H     -0.235378     -4.494660      4.626102
H     -1.373774     -3.499686      4.555437
O     -1.076594     -4.671628     -1.512103
H     -1.687104     -4.491151     -0.797580
H     -1.517391     -5.335114     -2.042504
O      1.101474      6.900695      0.905823
H      1.426844      6.019067      0.724981
H      0.867755      7.251147      0.046521
O     -2.543064     -5.847781      3.095191
H     -2.651951     -4.897141      3.078963
H     -2.012932     -6.015302      3.874125
```

[1]Bates, D. M.; Smith, J. R.; Tschumper, G. S. Efficient and Accurate Methods for the Geometry Optimization of Water Clusters: Application of Analytic Gradients for the Two-Body:Many-Body QM:QM Fragmentation Method to $(H_2O)_n$, $n = 3 - 10$. *J. Chem. Theory Comput.* **2011**, *7*, 2753–2760.
